# Ubiquitination dynamics in the early-branching eukaryote *Giardia intestinalis*

**DOI:** 10.1002/mbo3.88

**Published:** 2013-04-23

**Authors:** Carlos A Niño, Jenny Chaparro, Paolo Soffientini, Simona Polo, Moises Wasserman

**Affiliations:** 1 Laboratorio de Investigaciones Básicas en Bioquímica – LIBBIQ, Departamento de Química, Facultad de Ciencias, Universidad Nacional de ColombiaBogotá, Colombia; 2 Facultad de Ciencias Agrarias, Universidad de AntioquiaMedellín, Colombia; 3 IFOM, Fondazione Istituto FIRC di Oncologia MolecolareMilan, Italy; 4 Dipartimento di Scienze della Salute, Universita' degli Studi di MilanoMilan, Italy

**Keywords:** Differentiation, *Giardia*, proteasome, ubiquitination

## Abstract

Ubiquitination is a highly dynamic and versatile posttranslational modification that regulates protein function, stability, and interactions. To investigate the roles of ubiquitination in a primitive eukaryotic lineage, we utilized the early-branching eukaryote *Giardia intestinalis*. Using a combination of biochemical, immunofluorescence-based, and proteomics approaches, we assessed the ubiquitination status during the process of differentiation in *Giardia*. We observed that different types of ubiquitin modifications present specific cellular and temporal distribution throughout the Giardia life cycle from trophozoites to cyst maturation. Ubiquitin signal was detected in the wall of mature cysts, and enzymes implicated in cyst wall biogenesis were identified as substrates for ubiquitination. Interestingly, inhibition of proteasome activity did not affect trophozoite replication and differentiation, while it caused a decrease in cyst viability, arguing for proteasome involvement in cyst wall maturation. Using a proteomics approach, we identified around 200 high-confidence ubiquitinated candidates that vary their ubiquitination status during differentiation. Our results indicate that ubiquitination is critical for several cellular processes in this primitive eukaryote.

## Introduction

Modification by Ubiquitin (Ub) remodels target proteins by affecting their stability, interaction with other proteins, enzymatic activity, and also their subcellular localization. This versatile posttranslational modification regulates many cellular processes, including endocytosis, cell cycle, replication, and transcription (Geng et al. 2012; Komander and Rape 2012). Ubiquitin conjugation is mediated by an enzymatic cascade that includes the sequential action of ubiquitin-activating enzymes (E1), ubiquitin-conjugating enzymes (E2), and ubiquitin ligases (E3), resulting in a covalent linkage between the Ub carboxy-terminal glycine and the ε-amino group of a lysine (Lys) residue in the target protein (Husnjak and Dikic 2012). Ub can be conjugated as a single molecule (monoubiquitination), as several single Ub molecules (multimonoubiquitination) or as chains (polyubiquitination), where the first ubiquitin attached to the substrate serves as an acceptor for further cycles of ubiquitination. In this latter case, an isopeptide bond is formed between Gly76 of one ubiquitin and one of the seven potential lysines (K6, K11, K27, K29, K33, K48, or K63) present on the preceding ubiquitin (Komander and Rape 2012). Importantly, the ubiquitination process is highly dynamic and reversible due to the existence of specialized deubiquitinating enzymes (DUBs) able to remove Ub molecules from target proteins (Reyes-Turcu et al. 2009).

Mono- and multimonoubiquitination participate in proteasome-independent processes such as endocytosis, DNA repair, and gene expression regulation (Acconcia et al. 2009; Chen and Sun 2009; Komander and Rape 2012), whereas polyubiquitination is involved in both proteasome-dependent and proteasome-independent processes depending on the chain topology (Komander and Rape 2012). Ub chains linked through Lys48, Lys11, and Lys63 are the most abundant types in yeast, accounting for 29%, 28%, and 17%, respectively, of the total Ub chains (Xu et al. 2009). Lys48-poly-Ub chains are recognized by the proteasome, resulting in degradation (Komander 2009), whereas Lys11-poly-Ub participates in cell cycle regulation and endoplasmic reticulum-associated degradation (ERAD), as well as a proteasome degradation signal (Jin et al. 2008; Komander 2009). Lys63-poly-Ub chains, instead, have important roles in DNA repair, endocytosis, and signaling (Acconcia et al. 2009; Komander 2009).

The roles of ubiquitination are continuously growing, with evidence showing that ubiquitination participates in nearly every cellular process. With few exceptions (Chung et al. 2008; Ponts et al. 2011), the impact of ubiquitin in early unicellular eukaryotic organisms is instead almost ignored. Acquiring this knowledge would thus be a good opportunity to elucidate how Ub achieved its crucial role in the eukaryotic lineage. To explore the roles of ubiquitination in primitive eukaryotic organisms, we analyzed the process in the protozoa and early-branching eukaryote *Giardia intestinalis*.

*Giardia intestinalis* is a binucleated and flagellated gut parasite that infects humans and other vertebrates (Ankarklev et al. 2010). *Giardia intestinalis* has a compact genome (6470 ORFs, open reading frames) that encodes simple molecular machineries in comparison to other eukaryotes (Morrison et al. 2007). Additionally, *G. intestinalis* lacks classical organelles of higher eukaryotic cells, such as typical mitochondria, peroxisomes, and a Golgi apparatus (Ankarklev et al. 2010). In terms of phenotype, *G. intestinalis* has two structurally, functionally, and morphologically different stages: the binucleated and motile trophozoite (vegetative) and the quadri-nucleated cyst (infective). The cyst has a protective cell wall that enables it to resist harsh environmental conditions outside the host's intestine (Ankarklev et al. 2010). The life cycle begins when cysts are ingested from contaminated water or food. In the stomach, the cyst senses the low pH and begins excystation, which is finally completed in the upper small intestine where the trophozoites rapidly emerge and proliferate (Ankarklev et al. 2010). Some trophozoites are forced down with the intestinal flow and are induced to encyst (encystation) in the lower small intestine when they sense a low cholesterol concentration (Lujan et al. 1996). Mature cysts are then released in the feces (Ankarklev et al. 2010). Encystation involves important molecular and cellular processes such as DNA replication and nuclear division, as well as the synthesis, sorting, and transport of cyst wall components (Carranza and Lujan 2010).

The differentiation processes of excystation and encystation in *G. intestinalis* are models of primitive cellular adaptation systems and for this reason we used this organism as model system to understand and unravel the patterns and evolutionary mechanisms that Ub uses to shape the cells. In higher eukaryotes, Ub is expressed in the form of three different precursors: as a linear fusion protein consisting of five or more Ub copies, and as N-terminal Ub moieties fused to ribosomal proteins S27 and L40 (Catic and Ploegh 2005). In contrast, only one gene, GL50803_7110, encoding for a single Ub moiety has been identified in *G. intestinalis*. The Ub fusion to the S27a present in *G. intestinalis* represents a distant variant of Ub that is not cleaved and remains a structural component of the holoribosome (Catic et al. 2007). Constitutive expression of the single Ub gene has been detected at the mRNA level (Krebber et al. 1994; Gallego et al. 2007). Previously, we demonstrated a direct relationship between the ubiquitin-activating enzyme (E1) and *G. intestinalis* viability with regard to differentiation (Niño et al. 2012). Here, we analyzed the cellular implications of ubiquitination in *G. intestinalis* using a combination of biochemical, immunofluorescence-based, and mass spectrometry (LC-MS/MS) approaches. We present data indicating that ubiquitination is a dynamic and regulated event that participates in several cellular processes in *G. intestinalis*. Our results also suggest that ubiquitin-dependent proteasome functions are required for viable cyst formation.

## Experimental Procedures

### *Giardia intestinalis* culture and encystation

*Giardia intestinalis* WB/9B10 trophozoites (Carranza et al. 2002) were grown at 37°C in TYI-S-33 medium at pH 7.0 and were supplemented with 0.5 mg/mL bovine bile and 10% bovine serum (Keister 1983). Trophozoites were encysted in vitro according to the procedure described by Kane et al. (1991). Cells were collected at different times postinduction: trophozoites (0 h), encysting cells (6, 12, and 24 h) and cysts (48 h).

### Proteasome inhibition

To assess the effect of proteasome inhibition on cell viability and replication, trophozoites (1.2 × 10^6^ cells) were incubated for 48 h in the presence of proteasome inhibitors (1 μmol/L epoxomicin, 50 μmol/L MG132, and 10 μmol/L lactacystin, Boston Biochem). Once collected, the viability was determined by observing flagella movements in a Neubauer chamber. To assess the effect of proteasome inhibition on the ability of *G. intestinalis* to encyst, trophozoites were induced to encyst in presence of the proteasome inhibitors, and the number of cysts produced was counted. The viability of the cysts was determined by their ability to hydrolyze Fluorescein Diacetate (FDA), as previously described (Smith and Smith 1989), and was observed on a Zeiss Axiophot fluorescence microscope at 450–490 nm. All assays were performed twice and in triplicate.

### Immunoblot analysis

All protein extracts for immunoblotting were prepared freshly. Cells were resuspended and incubated for 1 h at 4°C in lysis buffer containing 50 mmol/L NaCl, 3 mmol/L MgCl_2_, 10 mmol/L 2-mercaptoethanol, 10% glycerol, 0.5% NP40, and a protease inhibitor cocktail (P8340-SIGMA, MO) in 30 mmol/L Tris (pH 7.5). Following centrifugation for 30 min at 8000*g* at 4°C, the supernatant containing the cytoplasm fraction was collected. The pelleted nuclei were resuspended and incubated for 1 h at 4°C in nuclei extraction buffer containing 400 mmol/L NaCl, 3 mmol/L MgCl_2_, 10 mmol/L 2-mercaptoethanol, 10% glycerol, 0.5% NP40, and P8340-SIGMA protease inhibitor cocktail in 30 mmol/L Tris (pH 7.5). The nuclear enriched fraction was collected by centrifugation at 8000*g* for 30 min at 4°C. The protein concentration was determined by the Bradford method (Bradford 1976). Samples were treated with loading buffer (Laemmli 1970), run on a 5–20% sodium dodecyl sulfate polyacrylamide gel electrophoresis (SDS-PAGE), and blotted onto a Polyvinylidene fluoride (PVDF) membrane (Immobilion P, Millipore, Billerica, MA). For the anti-Ub IB, membranes were treated in denaturing solution (6 mol/L guanidinium chloride, 20 mmol/L Tris pH 7.5, 1 mmol/L phenylmethanesulfonylfluoride (PMFS), and 5 mmol/L 2-mercaptoethanol) for 30 min at 4°C, followed by extensive washes with Tris-buffered saline and 0.1% Tween 20 (TBST) (Penengo et al. 2006). Membranes were blocked 1 h in TBST containing 5% Bovine serum albumin (BSA) and then incubated for 1 h with the corresponding antibody diluted in TBST containing 2% BSA. The following antibodies were used: P4D1 anti-Ub monoclonal antibody (1:1000, SantaCruz, Dallas, TX), anti-His_6_ tag monoclonal antibody (1:3000, Amersham, Hilden, CA), and anti-CWP1-TAMRA antibody (1:500, Waterborne Inc., New Orleans, LA). After primary antibody treatment, detection was performed with horseradish peroxidase-conjugated anti-mouse IgG and ECL™ (Amersham, CA).

### Immunofluorescence microscopy

Cells were fixed with 4% p-formaldehyde and permeabilized for 1 h at room temperature in phosphate buffered saline (PBS) containing 0.1% Triton X-100 and 10% donkey serum. Slides were incubated for 1 h at 37°C with the antibodies diluted in PBS. The following primary antibodies were used: rabbit polyclonal anti-gUb (generated by us using as antigen Ub purified protein from *Giardia*), mouse FK1 anti-Ub (1:200, Enzo Life Sciences, Plymouth Meeting, PA), which recognizes only poly-Ub proteins, mouse FK2 anti-Ub (1:200, Enzo Life Sciences), which recognizes both mono- and poly-Ub proteins but not Ub free; rabbit anti-Lys48-poly-Ub chains mAb-K48 (1:250, Millipore), and rabbit anti-Lys63-poly-Ub chains mAb-K63 (1:100, Millipore). Cells were then washed with PBS and incubated with the secondary antibody labeled with AlexaFluor 488 (1:100). The anti-CWP1-TAMRA antibody (1:100, Waterborne Inc.) was used for detecting the CWP1 protein (incubation for 1 h at 37°C), and the nuclei were stained with 4′,6-diamidino-2-phenylindole (DAPI). Slides were analyzed on an Olympus Upright BX51 fluorescence microscope.

### In vitro ubiquitination reaction

*Giardia intestinalis* trophozoites, encysting cells, and the cysts' NP40-extracts were used in the in vitro ubiquitination assay. The reaction mix contained 250 ng His_6_Ub (Sigma), 2 μmol/L Ub-aldehyde (Boston Biochem, Cambridge, MA), 20 μmol/L MG132 (Boston Biochem), 1.0 mmol/L ATP (Sigma), the ATP regeneration system (which consisted of 0.5 U phosphocreatine kinase [Sigma], 10 mmol/L creatine phosphate [Sigma], and 2.5 mmol/L MgCl_2_ [Sigma]), 10 mmol/L Tris-HCl (pH 7.5), and 50 μg of protein extract in a reaction volume of 15 μL. The reaction mix was incubated for 30 min at 25°C and halted by adding 5 μL of 4× loading buffer and incubating for an additional 10 min at 95°C. The His_6_Ub-tagged proteins were analyzed by IB with anti-His_6_-tag antibody (anti-His, Amersham) as previously described. Controls included reactions either without Ub-aldehyde and MG132 or without the ATP regeneration system.

### His_6_Ubiquitinated proteins purification and LC-MS/MS analysis

A reaction mixture containing 1000 μg of NP40-extracts and 5 μg of His_6_-Ub was incubated for 1 h at 25°C in a final volume of 300 μL. After the reaction, urea was added at a final concentration of 8 mol/L, and the reaction mixture was incubated with Niquel-Nnitrilotriacetic acid agarose (Ni-NTA) resin (Qiagen, Hilden, Germany) for 1 h at 4°C with shaking. The resin was collected by centrifugation (3000*g* for 2 min at 4°C) and washed ten times with washing buffer (8 mol/L urea, 5 mmol/L imidazole, 10 mmol/L Tris [pH 7.5]). To elute the bound ubiquitinated proteins (His_6_Ub-tagged proteins), the resin was incubated with 200 μL of elution buffer (8 mol/L urea, 500 mmol/L imidazole, 10 mmol/L Tris pH 7.5) for 15 min at 4°C with shaking. The suspension was centrifuged at 3000*g* for 5 min at 4°C, and the supernatant (His_6_Ub-tagged proteins) was collected carefully. To eliminate urea and imidazole, the proteins were precipitated with ethanol (90% final concentration), followed by resuspension in 30 μL of MS-sample buffer (50 mmol/L Tris-HCl [pH 6.8], 200 mmol/L dithiothreitol, 2% SDS, 0.1% bromophenol blue, and 10% glycerol), and separated by one-dimensional SDS-PAGE using a 4–12% polyacrylamide Nu-PAGE Novex Bis-tris gel (Life Technologies, Paisley, U.K.) with 1 mm thickness. After staining with Colloidal Blue staining kit (Invitrogen, CA), bands were cut from the gels and trypsinized as previously described (Shevchencko et al. 1996). Peptides were desalted as described (Rappsilber et al. 2003), dried in a Speed-Vac, and resuspended in 7 μL of 0.1% trifluoroacetic acid. LC-ESI-MS/MS of 5 μL of each sample was performed on a Fourier transformed-LTQ Ultra mass spectrometer (FT-LTQ Ultra, Thermo Electron, San Jose, CA). Peptide separation was performed on a linear gradient from 100% solvent A (5% acetonitrile, 0.1% formic acid) to 20% solvent B (acetonitrile, 0.1% formic acid) over 20 min, followed by 20% to 80% solvent B for 5 min at a constant flow rate of 0.3 μL/min on an Agilent chromatographic separation system 1100 (Agilent Technologies, Waldbronn, Germany), where the LC system was connected to a 10.5 cm fused-silica emitter with a 100 μm inner diameter (New Objective, Inc. Woburn, MA) and packed with ReproSil-Pur C18-AQ 3 μm beads (Dr. Maisch Gmbh, Ammerbuch, Germany) using a high-pressure pump loader (Proxeon, Odense, Denmark). Data acquisition mode was set to obtain one MS scan followed by five MS/MS scans of the five most intense ions from each MS scan. MS/MS spectra were limited to one scan per precursor ion followed by 1 min of exclusion. Mascot generic format (MGF) files were extracted using DTASuperCharge (v.1.19, http://www.cebi.sdu.dk), and a database search was performed using Mascot Daemon set up with the following parameters: database, NCBI–nr; taxonomy, all entries; enzyme, trypsin; maximum missing cleavage, 2; fixed modification, carbamidomethyl (C); variable modification, oxidation (M); peptide tolerance, 10 ppm; MS/MS tolerance, 0.5 Da; and instrument, ESI-TRAP (Thermo Electron, San Jose, CA). Two biological replicates were performed and filtering analysis and manual curation of mass spectrometry data were performed. All the proteins identified in the reaction and present in the control (nonspecific retention) were discarded.

LTQ-Orbitrap RAW files were analyzed with DTA Supercharge (v.1.19, http://www.cebi.sdu.dk), yielding extracted fragmentation spectra in MGF format. Database search was performed using Mascot Deamon or Proteome Discoverer set up with the following parameters:

Mascot v.2.3.2: Database NCBInr, Taxonomy Eucaryotes, enzyme Trypsin, Max missing cleavage 4, fixed modification none, variable modification oxidation (M), GlyGly (K), Leu Arg Gly Gly (k), peptide tolerance 10 ppm, MS/MS tolerance 0.5 Da, Instrument ESI-TRAP.

Proteome Discoverer (1.1.0.263 Thermo Fisher Scientific Inc., Waltham, MA): MS/MS spectra were analyzed using Sequest Sequest v.1.12, Database *Giardia*DB-2.5 (http://www.giardiadb.org), enzyme Trypsin, Max missing cleavage 4, fixed modification none, variable modification oxidation (M) +15.995 Da, GlyGly (K) +114.043 Da, LeuArgGlyGly (K) +383.228 Da, peptide tolerance 10 ppm, MS/MS tolerance 0.5 Da. All Mascot or Sequest ubiquitined peptide hits were validated manually.

### Purification of endogenous ubiquitinated proteins

Cell lysates (500 μg) supplemented with 10 mmol/L N-Ethylmaleimide (NEM) and 20 μmol/L MG132 were incubated with the mouse FK2 anti-Ub (Enzo Life Sciences) or anti-Lys48-poly-Ub chain antibody (mAb-K48, Millipore) for 1 h at 4°C with shaking. Then, protein A-sepharose CL4B (GE Healthcare Life Sciences, Pittsburgh, PA) was added, and the mixture was incubated for 1 h. The beads were washed 10 times with PBS, and the proteins were eluted by treatment with Laemmli buffer for 10 min at 95°C. The proteins were separated by one-dimensional SDS-PAGE using a 4–12% polyacrylamide Nu-PAGE Novex Bis-tris gel with 1 mm thickness. After staining with Colloidal Blue staining kit (Invitrogen, CA), bands were cut from the gels and trypsinized as previously described. Mass spec analysis was performed as previously described.

## Results and Discussion

### Ubiquitination is an active and regulated process in *G. intestinalis*

**Figure 1:**
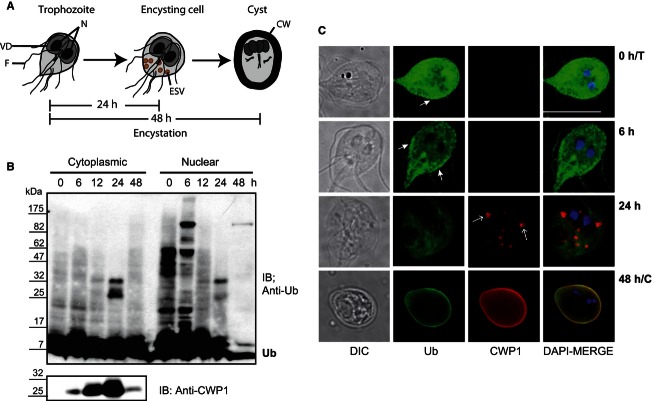
Ubiquitination is a dynamic process in *Giardia*. (A) Schematic representation of the in vitro encystation process. Trophozoites were induced to encyst and after 48 h cyst were obtained. Principal organelles are indicated as N: nucleus, VD: ventral disc, F: flagella, ESV: encystation-specific vesicles, and CW: cyst wall. (B) Immunodetection of Ub and Ub-protein conjugates. Trophozoites were induced to encyst, and cells were collected at different times during the process (0, 6, 12, 24, and 48 h). Extracts of different encystation stages were subjected to SDS-PAGE and immunoblotted with monoclonal anti-Ub. Immunoblotting detection of CWP1 (26 kDa) was used as the encystation marker. (C) Immunolocalization of Ub and Ub-protein conjugates. Cells were collected, fixed with p-formaldehyde, and used for immunofluorescence analysis. Ub and Ub-conjugated proteins were detected with anti-gUb. Anti-CWP1-TAMRA was used to detect CWP1 protein. Nuclei were stained with DAPI. The different stages are indicated as follows: (0 h/T) trophozoites, (6 h) encysted cell 6 h postinduction, (24 h) encysted cell 24 h postinduction, and (48 h/C) cyst obtained after 48 h of induction. Full arrows indicate the peripheral spots. Open arrows indicate encystation-specific vesicles (ESVs). Scale bar is 10 μmol/L. DIC is differential interference contrast microscopy.

Previously, we showed that the ubiquitin-activating enzyme gene (*e1*) is essential for *Giardia* viability, and we suggested a role for ubiquitination in this parasite (Niño et al. 2012). To understand the observed lethality of *e1* silencing, we explored whether ubiquitination is an active process in *Giardia* and whether it plays a role during its differentiation. To this end, we analyzed the changes in expression and localization of Ub and Ub-protein conjugates in trophozoites and during encystation (Fig. 1).

Ub and Ub-protein conjugates were easily detected by immunoblotting (IB, P4D1 antibody, Fig. 1B) and immunofluorescence (IF, gUb antibody, Fig. 1C) in trophozoites, encysting cells, and cysts, revealing an active ubiquitination system in *Giardia*. As encystation is a process in which an active metabolic trophozoite transitions into an inactive cyst, it is easy to envision that degradation of several proteins might be required during the process. Indeed, we observed clear differences in the pattern and cellular distribution of ubiquitinated proteins at the different life stages, both in the cytoplasm and in the nucleus (Fig. 1). Trophozoites, which are the motile and replicative stage of *Giardia*, showed good level of Ub-protein conjugates (Fig. 1B, time 0), with a wide distribution throughout the entire cell and enhanced localization close to the plasma membrane (Fig. 1C, full arrows). Ubiquitination was markedly affected by encystation induction: at 24 h upon encystation induction ubiquitination is limited to few bands (Fig. 1B), with a diffuse pattern in the cytoplasm (Fig. 1C). Intriguingly, Ub signal distribution does not correlate with the encystation-specific secretory vesicle system (ESVs) (Fig. 1C, open arrows) that transports the cyst wall proteins (CWPs) to the cellular periphery (Carranza and Lujan 2010) as no colocalization is evident with the wall protein CWP1 (Fig. 1C, 24 h), whose expression peaks between 12 h and 24 h upon induction (Fig. 1B, lower panel). In the cysts, Ub-protein conjugates were localized almost exclusively in the cyst wall (Fig. 1C, time 48 h), similarly to the observed localization of the E1 enzyme in cysts (Niño et al. 2012) and of proteasomal subunit Giα7 (Stefanic et al. 2006).

### Different ubiquitin modifications show specific cellular distribution and modulation during encystation

**Figure 2:**
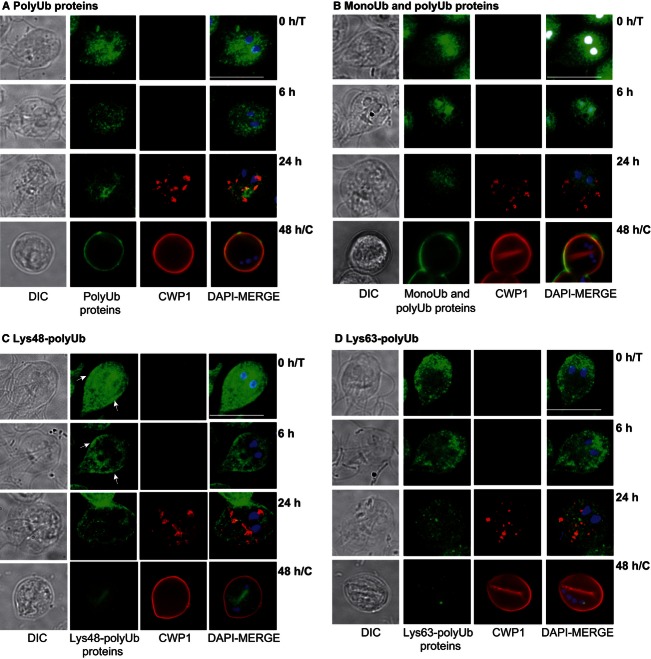
Changes in the distribution of mono- and polyubiquitinated proteins during encystation. Trophozoites, encysting cells, and cysts were collected, fixed with p-formaldehyde, and used for immunofluorescence analysis. (A) FK1 antibody recognized polyubiquitinated proteins. (B) FK2 antibody recognized mono- and polyubiquitinated proteins. (C) Anti-Lys48-poly-Ub chains (mAb-K48) recognized Lys48-polyubiquitinated proteins. Full arrows indicate the peripheral spots. (D) anti-Lys63-poly-Ub chains (mAb-K63) recognized Lys63-polyubiquitinated proteins. Anti-CWP1-TAMRA was used to detect CWP1 protein, and nuclei were stained with DAPI. The different stages are indicated as follows: (0 h/T) trophozoites without induction; (6 h) encysted cell 6 h postinduction; (24 h) encysted cell 24 h postinduction; and (48 h/C) cyst obtained after 48 h of induction. Scale bar is 10 μmol/L. DIC is differential interference contrast microscopy. Staining and time points are as in Figure 1.

We then performed the same analysis with a set of more specific antibodies against Ub. To discriminate between mono- and polyubiquitinated proteins, we performed the IF analysis with FK1 and FK2 antibodies (Fig. 2A and B). As FK2 recognizes both mono- and polyubiquitinated proteins but not free ubiquitin and FK1 only polyubiquitinated proteins, we ascribed the FK2 signals that were absent with FK1 as corresponding to monoubiquitinated proteins. In trophozoites, signals in cytoplasm were observed with both antibodies (Fig. 2A and B, time 0 h). Polyubiquitinated proteins were distributed as discrete spots in the cytoplasm, with no (observed) signal in the nucleus (Fig. 2A). Following the induction of encystation, they remained distributed as spots with a decrease in the signal intensity (Fig. 2A and B, time 24 h). Conversely, an intense fluorescent signal in the nucleus was observed only with the FK2 antibody (Fig. 2B, time 0 h), suggesting that the nuclear signal shown in Figure 1B is mainly due to monoubiquitinated proteins. Of note is that the nuclear signal persisted at 6 h postinduction of encystation and was weaker in late encysting cells (24 h), suggesting deubiquitination or even degradation of the nuclear monoubiquitinated proteins in the late stages of encystation. Confirming previous data, signals for both mono- and polyubiquitinated proteins were observed almost exclusively at the level of the cell wall in the cysts.

Next, we analyzed the performance of specific Lys48- and Lys63-antibodies, which are able to recognize polyubiquitinated proteins destined for proteasomal degradation and for trafficking, respectively (Fig. 2C and D). Lys48-polyubiquitinated proteins were distributed throughout the entire trophozoite with a greater presence close to the nucleus and at the plasma membrane (Fig. 2C, full arrows). In the encysting cells, the signal intensity decreased, and 24 h after induction the localization pattern changed to a regular spot distribution inside the cell and around the membrane (Fig. 2C, 24 h). Lys63-polyubiquitinated proteins were detected in trophozoites, encysting cells and cysts, and their localization pattern was assessed to discrete spots (Fig. 2D). Again, no colocalization was evident with CWP1 marker, arguing against a role of Ub in the trafficking of ESVs of *Giardia*. With both antibodies at the cyst stage, the signal is limited to few spots in the interior of the cell and almost excluded from the cell wall (Fig. 2C and D, time 48 h). These unexpected results suggest a role for atypical Ub chains in the biogenesis or maturation of the cyst wall and deserves further investigation.

### Ubiquitin proteasome-dependent functions are not required for trophozoite replication but are important for cyst viability

**Figure 3:**
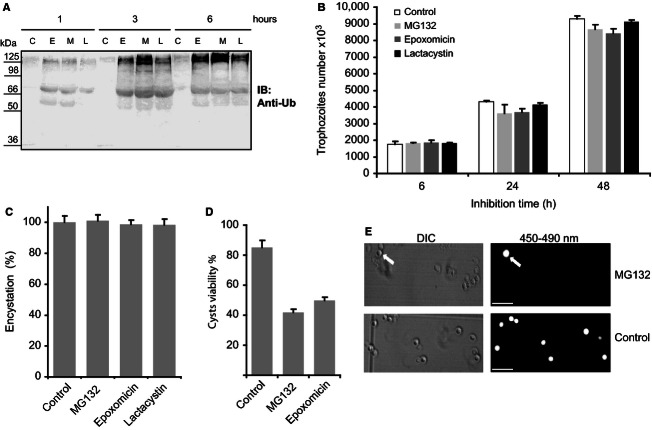
Proteasome inhibition affects cyst viability. (A) Detection of Ub-protein conjugates under proteasome inhibition. Trophozoites were incubated in the presence of DMSO as a control (C), 1 μmol/L epoxomicin (E), 50 μmol/L MG132 (M), and 10 μmol/L lactacystin (L). Parasites were harvested at 1, 3, and 6 h after inhibitor was added. The samples were subjected to SDS-PAGE and IB with anti-Ub. (B) Effect of proteasome inhibition on trophozoite replication. Trophozoites were incubated in the presence of the proteasome inhibitors epoxomicin (1 μmol/L), MG132 (50 μmol/L), and lactacystin (10 μmol/L). Cells were harvested at 6, 24, and 48 h after inhibition. The number of viable trophozoites is shown as the mean of two experiments in triplicate ±SD. (C) Effect of proteasome inhibition on encystation. Trophozoites were induced to encyst in presence of the proteasome inhibitors epoxomicin (1 μmol/L), MG132 (50 μmol/L), and lactacystin (10 μmol/L); after 48 h cyst were collected and counted. (D) Effect of proteasome inhibition on cyst viability. The viability of the cysts treated with either epoxomicin (1 μmol/L) or MG132 (50 μmol/L) was determined by incubation with FDA followed by evaluation of its intracellular hydrolysis by fluorescence microscopy at 450–490 nm. The percentage of viable cysts is shown as the mean of two experiments in triplicate ±SD. (E) Micrographs of cysts after treatment with 50 μmol/L MG132. Cysts were analyzed by incubation with FDA dye. The arrow shows the only one viable cyst observed in this field. Controls were treated with DMSO only. Scale bar is 20 μmol/L. DIC, differential interference contrast microscopy; FDA, fluorescein diacetate; IB, immunoblotting.

Prompted by these results, we investigated whether Ub proteasome-dependent processes are involved in replication and differentiation in *Giardia*. Blocking the proteasome impairs cell viability in most eukaryotes, including protozoan parasites (Shaw et al. 2000; de Diego et al. 2001; Makioka et al. 2002), as opposed to prokaryotic cells (Ruepp et al. 1998; De Mot et al. 1999; Voges et al. 1999). To analyze the Ub proteasome-dependent functions in *G. intestinalis*, we evaluated the effects of proteasome inhibition on the replication, viability, and differentiation of trophozoites. Treatment with various inhibitors resulted in an accumulation of Ub-protein conjugates (Fig. 3A), indicating that the chemical inhibitors penetrated the cell and inhibited peptidase activity, as observed in other eukaryotic cells (reference). However, although the concentrations used are in the range of cytotoxicity for mammalian cells, the treatment did not affect either trophozoite replication or viability (Fig. 3B). After 48 h, approximately five replication events, trophozoites in the presence of proteasome inhibitors grow similar to the control (Fig. 3B). When proteasome inhibition was performed during encystation, we did not observe any effect on total cyst production (Fig. 3C). This result is consistent with previous observations of an unaffected encystation rate in *Giardia* when the proteasome is inhibited (Stefanic et al. 2006) and suggests that degradation of the proteins is not essential for *Giardia* differentiation. Nonetheless, we measured a significant reduction (∼50%) in cyst viability (Figs. 3D, 5E), suggesting that in the absence of the proteasome the cyst wall is not organized properly.

### Purification and identification of Ub-protein conjugates

**Figure 4:**
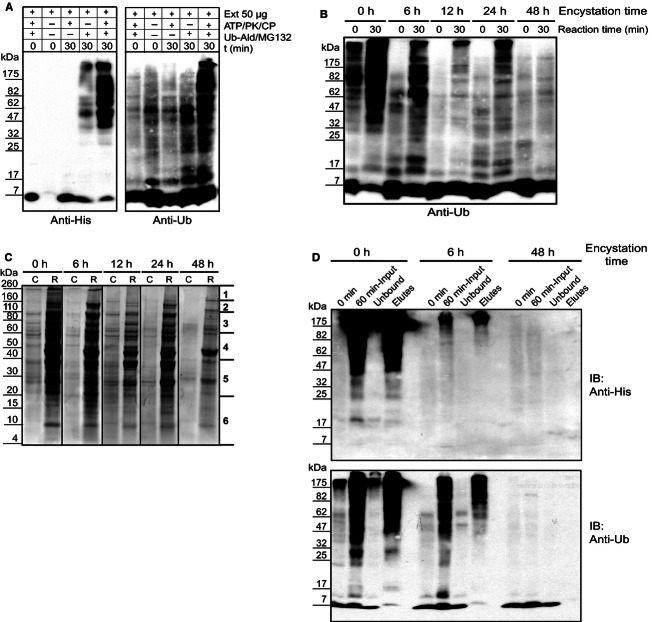
In vitro ubiquitination assay and purification of His_6_-Ubiquitinated proteins with *Giardia* extracts. (A) In vitro ubiquitination reaction using trophozoite extracts. Reactions were performed for 30 min at 25°C with or without inhibitors (MG132 and Ub-aldehyde) and with or without the ATP-regenerating system, as indicated. (B) In vitro ubiquitination reaction using representative extracts of different encystation stages. Reactions were performed as in A. (C) A large scale in vitro ubiquitination assay was done / performed and His_6_Ub-protein conjugates were purified by affinity chromatography (Ni-NTA) under denaturing conditions. Purified His_6_Ub-protein conjugates were separated by SDS-PAGE and stained with colloidal Coomassie Blue; then each lane of the gel was excised, digested with trypsin, and analyzed by LC-MS/MS. (D) Approximately 15 μL aliquots from the different steps of the purification process were evaluated by immunoblotting with anti-His tag and anti-Ub antibodies. The lanes were marked as follows: 0 min, initial mix reaction; 60 min input, incubation at 25°C for 60 min; unbound, unbound proteins; elutes, proteins that eluted with 500 mmol/L Imidazole.

To determine which cellular processes in *Giardia* involve ubiquitination, we set to purify and identify ubiquitinated proteins during the various differentiation steps. The identification of the ubiquitinated proteins is often troublesome because of their low abundance, lability, and rapid proteasome-mediated degradation. Indeed, when we tried to perform immunoprecipitation of endogenously ubiquitinated proteins we scored a faint signal in the anti-Ub blot that prevented further mass spectrometry analysis (Fig. S1). To have a better controlled system, we set up an in vivo/in vitro ubiquitination assay where only His6Ub-protein has been added to the extract in order to purify ubiquitinated conjugates by affinity chromatography (Ni-NTA) under denaturing conditions. It is important to note that the sole enzymes present in the reaction are E1, E2s, and E3s endogenously present in the *Giardia* extract, while the energy that is required for the process is provided by an ATP regeneration system (see methods for details). The assay of *Giardia* extracts is performed in the presence of proteasome and DUBs inhibitors, which allowed for maximal recovery of ubiquitinated substrates. First, we tested the in vivo/in vitro ubiquitination assay using trophozoite extracts. The His_6_Ub-protein conjugates were detected by IB with an anti-His and anti-Ub antibody (Fig. 4A). Detection of high-molecular weight His_6_Ub signals confirmed that the exogenous His_6_Ub was conjugated to proteins. The efficiency of the reaction was substantially reduced when the ATP regeneration system was lacking and abrogated in the absence of proteasome and DUBs inhibitors, validating the specificity of the reaction (Fig. 4A). The assays were then performed with extracts of encysting cells (6, 12, and 24 h postinduction) and cysts (48 h postinduction). Similar to what we observed in the Ub-IB (Fig. 1B), the highest level of His_6_Ub-protein conjugates was detected in trophozoites with a reduction of these products once encystation was induced (Fig. 4B). Thus, also in a controlled in vivo/in vitro system, a decrease in the amount of ubiquitinated proteins is visible during encystation. These results suggest that the observed reduction is not due to increased degradation or deubiquitination (since proteasome and DUBs inhibitors are present) but rather to different availability of the substrates or different activity of the ubiquitinating enzymes at various stages of the differentiation process. It should be noted that our in vivo/in vitro assay cannot account for a different localization of enzymes versus substrates due to the destruction of the organelles during protein extraction.

**Figure 5:**
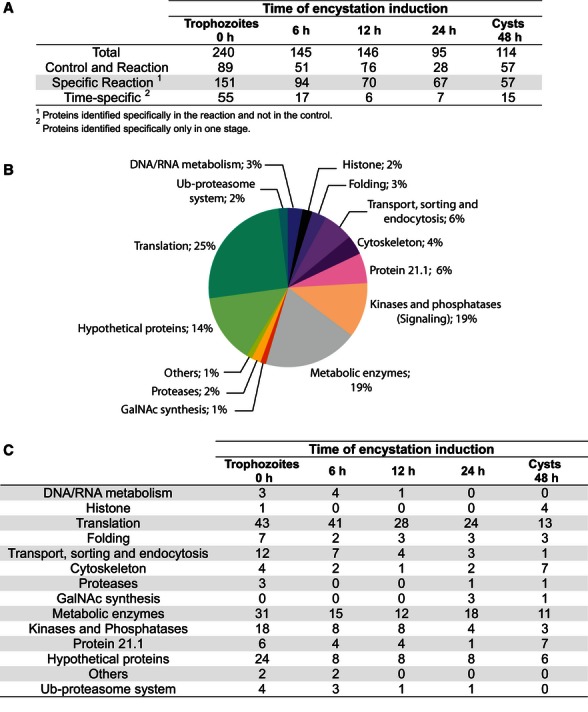
Results and functional classification of the ubiquitinated proteins identified by LC-MS/MS. (A) Results summary of ubiquitinated proteins identified by LC-MS/MS. (B) Ubiquitinated proteins were classified into different groups according to their annotations in *Giardia*DB (http://Giardiadb.org/) and NCBI. Each category is expressed as a percentage of the total identified proteins. (C) Distribution of ubiquitinated proteins in the different encystation stages.

Our ultimate goal was the identification of the ubiquitinated substrates in trophozoites, encysting cells, and cysts. Tagging the ubiquitinated proteins with exogenous His_6_Ub enabled us to purify them under denaturing conditions favoring the identification of covalently ubiquitinated proteins over the Ub-associated proteins (Peng 2008; Argenzio et al. 2011). Thus, we set up a large scale in vivo/in vitro ubiquitination assay where only His_6_Ub-protein has been added to the extract in order to purify ubiquitinated conjugates by affinity chromatography (Ni-NTA) under denaturing conditions. Purified His_6_Ub-protein conjugates were separated by SDS-PAGE and stained with colloidal Coomassie Blue; each lane of the gel was then excised, digested with trypsin and analyzed by LC-MS/MS (Fig. 4C). Chromatography was simultaneously performed with the same amount of extracts to filter out proteins that were nonspecifically retained in the resin (discarded from the final list, Table S2). Two biological replicates were performed, an example of which (with extracts from cells 0, 6, and 48 h after induction of encystation) is shown in Figure 3D. Remarkably, 74% of proteins were identified in both experiments, indicating a high level of reproducibility. Filtering analysis and manual curation of the data were performed, and the final results (corresponding to the merge between the two biological replicates) are summarized in Figure 5A and detailed in Table S1.

A total of 211 proteins (∼3.2% of *Giardia*'s proteome) were specifically identified with our approach as ubiquitinated candidates. Ubiquitination substrates were more abundant in trophozoites with 151 proteins, 55 of which were identified only in this stage. In agreement with IB and IF analysis, once encystation was induced, the number of ubiquitinated substrates decreased with 94 at 6 h, 70 at 12 h, 67 at 24 h postinduction, as well as 57 proteins identified in the cysts (15 of which were identified exclusively in this stage). The identification of the only *bona fide* ubiquitinated protein known so far in *Giardia*, the glucosamine-6-phosphate deaminase protein, GNP (Lopez et al. 2002), demonstrated that the strategy was effective. In addition, we identified nine ubiquitination sites in nine different target proteins (Table S4). Finally, a large number of the identified substrates corresponds to proteins previously found to be ubiquitinated in other organisms (see for example Peng et al. 2003; O'Reagan et al. 2007; Daulny et al. 2008; Koiwai et al. 2008; Argenzio et al. 2011; Ponts et al. 2011), suggesting that, also in *Giardia*, ubiquitination plays important roles in translation/ribosome function, metabolic pathways, endocytosis, and signaling. To further validate our in vivo/in vitro assay, as a proof of principle, we performed mass spectrometry analysis on the trophozoite extracts immunoprecipitated with the FK2 or the Lys48-poly-Ub anti-Ub antibody (Fig. S2A and B). We identified 17 and 40 proteins, respectively, with a significant overlap with the in vivo/in vitro dataset (Table S5). Interestingly, in both datasets, we were able to identify the native linkage-specific peptide for Lys48 chains generated upon trypsin digestion (Fig. S2C and Table S4).

### Functional classification of ubiquitinated substrates reveals participation of ubiquitination in diverse cellular processes in *Giardia*

The identified proteins were classified into 14 functional categories based on their annotations in *Giardia*DB (http://giardiadb.org) and NCBI (Fig. 5B). Strikingly, variations during encystation were observed not only in the total number of identified ubiquitination substrates but also in the distribution of their functional group (Fig. 5C). Groups such as translation, DNA/RNA metabolism, transport/sorting/endocytosis, folding, enzymes, highly present in trophozoites, showed a reduced incidence during encystation and in the cyst. On the contrary, groups such as cytoskeletal proteins and the protein 21.1 family presented a higher number of proteins in the cysts compared with the trophozoites. To identify domains and protein homologs, we also performed analyses by BLAST, Pfam, and SMART on three interesting groups: kinases, the protein 21.1 family, and hypothetical protein sequences (Tables S3).

During encystation, CWPs are expressed and transported to form the cyst wall together with N-acetyl-galactosamine polysaccharide GalNAc (Adam 2001). The enzymes that participate in the biosynthesis of GalNAc are specifically expressed when encystation is induced (Lopez et al. 2003). Three of these enzymes were identified in our mass spectrometry analysis: the previously cited GNP, glucose-6-phosphate N-acetyltransferase (GNA), and phosphoacetylglucosamine mutase (AMG). GNP was identified at 24 h postinduction of encystation and in cysts, in agreement with the previous report on the in vivo ubiquitination of GNP (Lopez et al. 2002). GNA and AMG were identified as ubiquitinated 24 h after encystation induction. These findings strongly indicate that ubiquitination, possibly different from the classical Lys48-mediated, participates in the GalNAc biosynthesis and in the formation of the wall matrix.

For the first time, we identified the four histones of Giardia as ubiquitinated: H2A alone in trophozoites and all four histones (H2A, H2B, H3, and H4) in cysts (*Giardia* does not have H1). Histone modification in *Giardia* has not been documented previously except for a report related to histone acetylation and its possible role in differentiation (Sonda et al. 2010). The monoubiquitination of H2A (uH2A) is one of the most abundant histone modifications in mammalian cells (5–15% of total H2A), important for transcriptional repression and the maintenance of the genome's integrity (Osley et al. 2006; Vissers et al. 2008). H2B ubiquitination is required for the recruitment and stabilization of the double-strand breaks forming machinery possibly important for recombination events (Yamashita et al. 2004). Indeed, it has been proposed that nuclear fusion and homologue recombination occur only in the cyst (Poxleitner et al. 2008). Through bioinformatics analysis, we have also identified the E3-ligase responsible for H2B ubiquitination, namely Bre1 (Hwang et al. 2003), in the protein 21.1 XP_001709481.1 (Table S3), suggesting that the molecular machinery for H2B ubiquitination is already present in this early-branching eukaryote. It will be interesting to investigate whether the difference between trophozoites and cysts in terms of histones ubiquitination is correlated to the changes observed in the transcriptome between these two stages (Faghiri and Widmer 2011).

Kinases are important cell cycle modulators, and several of them are degraded by the Ub-proteasome system (Lu and Hunter 2009). Our bioinformatics analysis of the group of kinases allowed us to identify some relevant domains in their sequences. Five NEK kinases were identified here as ubiquitinated substrates in trophozoites. NEK kinases are serine/threonine kinases related to Never In Mitosis gene A (NIMA) kinases. In yeast, NIMA kinase Fin1 has multiple roles in mitotic progression, chromatin condensation, and cytokinesis (O'Reagan et al. 2007) and is regulated by ubiquitin-mediated degradation through the anaphase-promoting complex (APC/C) E3-ligase (Woodbury and Morgan 2007). Similarly, Polo-like kinase 1 (Plk1) regulates the entrance and exit to mitosis, and is essential for cytokinesis (Archambault and Glover 2009), and is degraded by the proteasome via the APC/C complex. We identified two Polo Box domains in the PLK kinase (XP_001705776, ubiquitinated substrate in trophozoites). Interestingly, we did not recognize any APC/C components in the genome of *Giardia* with our bioinformatics analysis. What logically follows from these data is the possibility that a different regulation occurs in this ancestral eukaryote with a divergent E3 ligase responsible for the ubiquitination of these kinases.

Another interesting class (6% of the proteins identified) is one of the proteins involved in transport, sorting, and endocytosis, such as dynamin, clathrin heavy chain, and the AP-2α subunit. These proteins were previously localized in the trophozoite peripheral vacuoles (PVs) (Gaechter et al. 2008; Rivero et al. 2010) whose function as lysosomes and early- and late endosomes (Lanfredi-Rangel et al. 1988; Lujan and Touz 2003). Whether the anti-Ub signal present at the periphery of the cell membrane (Figs. 1C, 3A and B) is due to ubiquitination of the endocytic proteins and whether there is a relationship between ubiquitination and PVs similar to that in higher eukaryotes (MacGurn et al. 2012) are intriguing questions that deserve further investigations. Of note, variant-specific surface protein 9B10 (VSP-9B10) was identified as an ubiquitinated substrate 24 h after encystation induction and in cysts. VSPs are related to the antigenic variation process that enables the parasite to evade the host's immune response (Prucca et al. 2011) and are localized in PVs late in encystation and in the cyst (Svärd et al. 1998). How they are transported and whether they are degraded in these vacuoles is not known. Indeed, Ub-mediated internalization of the invariant surface glycoproteins (ISGs) has been reported recently in *Trypanosoma brucei* (Chung et al. 2008; Leung et al. 2011), another protozoan, strongly suggesting that the endocytic role of ubiquitin is an ancestral function exerted by this posttranslational modification.

Taken together, our data strongly support the notion that ubiquitin plays already multiple roles in the biology of this early-branching eukaryote. Based on this study, we would like to propose *Giardia* as a valuable model (especially now that its genome sequence has been completed) to investigate and understand the ubiquitination system in evolutionary terms. In this line, we have undertaken a bioinformatic analysis to identify components of the ubiquitination machinery (Moises Wasserman, paper in preparation). In addition to the single E1 that we have recently characterized (Niño et al. 2012), we identified 12 E2 enzymes, 67 E3 ligases (5 HECT ligases and 62 RING ligases), and 12 DUBs that await further characterization. Of note, two of the *Giardia* HECT enzymes show clear homology with mammalian Nedd4 and E6AP known as Lys63- and Lys48-specific enzymes, respectively (Fig. S3).

It remains to be established whether the ubiquitination of candidate proteins, uncovered herein, serves to regulate their degradation or has other, yet to be discovered, nonproteolytic functions. Indeed, the kind of ubiquitin modification and the ultimate functional significance of the modification at the single protein level remain to be elucidated. Nonetheless, our results are a first step in the identification of new therapeutic targets that could form the basis for novel strategies of intervention against this parasite that evades the host's immune response and produces chronic and recurrent infections.

## Supporting Information

**Figure S1.** Purification of Ubiquitinated proteins and specific Lys48-polyUb proteins by immunoprecipitation. Cell Lysates of different encystation stages (0, 6, 12, 24, and 48 h) were prepared. Lysates (500 μg) supplemented with 10 mmol/L NEM and 20 μmol/L MG132 were incubated for 1 h with anti-Ub FK2 antibody or anti-Lys48-polyUb chain antibody. Immunoconjugates wre purified with protein A-SepharoseCL4B (GE HealthcareLife Sciences) and analyzed by immunoblotting with P4D1 anti-Ub monoclonal antibody. Fractions of different immunoprecipitation stages are indicated, Input: initial lysate, NB: not binding fraction, IP: immunoprecipitated, and LW: last wash.

**Figure S2.** Validation strategy, in vivo purification of ubiquitinated species from trophozoite extracts. Endogenous ubiquitinated proteins were purified by immunoprecipitation with anti-Ub FK2 antibody or anti-Lys48-polyUb chain antibody. (A) The ubiquitinated purified proteins were separated by SDS-PAGE and after staining the gel was cut and analyzed by HPLC-MS/MS. (B) A fraction of immunoprecipitated proteins was analyzed by immunobloting with P4D1 anti-Ub monoclonal antibody. Fractions of different immunoprecipitation stages are indicated, IP: immunoprecipitated, Input: initial lysate, NB: not binding fraction, and LW: last wash. (C) MS/MS spectrum of the linkage-specific peptide for Lys48 chains (signature peptide) with the Gly-Gly modified lysine residue. Peptide sequence is shown with the annotation of the identified matched b ions in red and the y ions in blue.

**Figure S3.** Sequence alignment of the HECT domains of human Nedd4 family members, E6AP, yeast R sp5, and two putative Giardia HECT (XP_001704118.1 and XP_0017006167.1), performed with Muscle. Sequences are color-coded according to sequence conservation. Uniprot accession codes qre provided with the protein names.

**Table S1.** Proteins identified with the in vitro ubiquitination assay.

**Table S2.** Proteins presents in both control and in vivo/in vitro ubiquitination assay.

**Table S3.** Sequence analysis of protein kinases, protein 21.1 and hypothetical proteins identofied with the in vivo/in vitro ubiquitination assay.

**Table S4.** Ubiquitinations sites identified in the in vitro/in vivo analysis.

**Table S5.** Edogenous proteins from trophozoites purified by immunoprecipitation.
