# Supplementary material for: Ubiquitination dynamics in the early-branching eukaryote Giardia intestinalis
Source: Microbiologyopen. 2013 Apr 23;2(3):525–39. doi: 10.1002/mbo3.88 (PMC3684764; doi:10.1002/mbo3.88)
Supplement: Supplementary file 4 [file mbo30002-0525-SD4.pdf]

Supplementary Table 1
Proteins identified with the *in-vitro* ubiquitination assay

			Trophozoites								Cyst	
**Encystation induction time**			**0 hours**		**6 hours**		**12 hours**		**24 hours**		**48 hours**	
**Accession number**	**Mass**	**Description**	Peptides	Mascot score	Peptides	Mascot score	Peptides	Mascot score	Peptides	Mascot score	Peptides	Mascot score
**DNA/RNA Metabolism (Replication, segregation, transcription)**												
XP_001708867	238596	RNA polymerase large subunit Rpb1	ND		2	119	ND		ND		ND	
XP_001708583	144869	DNA-directed RNA polymerase RPB2 (RNA polymerase beta subunit)	6	274	2	92	ND		ND		ND	
XP_001706594	105079	Transcription factor, putative	2	99	ND		ND		ND		ND	
XP_001708536	35203	Fibrillarin-like pre-rRNA processing protein Narcisi et al	3	137	ND		ND		ND		ND	
XP_001709373	55296	Nucleolar protein NOP2 (methyltransferase)	ND		2	113	ND		ND		ND	
XP_001707973	109178	Spindle pole protein, putative (chromosome segregation protein SMC)	ND		2	163	ND		ND		ND	
XP_001705612	220913	5'-3' exoribonuclease 2	ND		ND		2	97	ND		ND	
**Histone**												
XP_001704715	13848	Histone H2A	2	150	ND		ND		ND		9	603
ABV60223	12398	histone H2B	ND		ND		ND		ND		9	573
XP_001707235	16292	Histone H3	ND		ND		ND		ND		3	198
ABV60243	8773	histone H4	ND		ND		ND		ND		6	413
**Translation**												
BAA06215	90464	elongation factor 2 (EF2)	11	620	11	558	2	143	ND		ND	
XP_001707321	45169	Elongation factor 1-gamma (EF1gamma)	2	125	ND		ND		ND		ND	
XP_001704069	24724	Translation elongation factor (EF1beta)	2	150	ND		ND		ND		ND	
XP_001708522	70231	Arginyl-tRNA synthetase	2	124	6	352	ND		ND		ND	
XP_001705722	62541	Aspartyl-tRNA synthetase	5	291	11	703	ND		ND		ND	
XP_001705007	87528	Glutaminyl-tRNA synthetase	6	362	ND		ND		ND		3	155
XP_001707446	80061	Glutaminyl-tRNA synthetase	ND		9	538	ND		2	124	ND	
XP_001707384	65910	Lysyl-tRNA synthetase	3	167	ND		ND		ND		ND	
XP_001705100	78963	Threonyl-tRNA synthetase	5	352	2	75	ND		ND		2	96
XP_001709365	139119	Valine-tRNA ligase	ND		2	63	ND		ND		ND	
XP_001706205	74998	RNase L inhibitor (Ribosome maturation and Translation)	2	98	ND		ND		ND		ND	
XP_001705172	204027	RRNA biogenesis protein RRP5 (Ribosomal biogenesis)	9	419	3	153	ND		ND		ND	
XP_001704254	27002	Ribosomal protein L2	4	230	5	257	ND		ND		ND	
XP_001705236	42596	Ribosomal protein L3	9	546	11	771	4	212	3	164	2	118
XP_001708810	34947	Ribosomal protein L4	10	656	7	469	5	382	2	126	ND	
XP_001706234	33856	Ribosomal protein L5	2	100	ND		ND		ND		ND	
XP_001705231	27023	Ribosomal protein L7	6	361	9	585	3	154	ND		ND	
XP_001706321	25348	Ribosomal protein L7a	4	272	6	346	4	296	3	175	ND	
XP_001705797	20823	Ribosomal protein L9	11	648	9	529	ND		8	502	2	165
XP_001707575	24963	Ribosomal protein L10a	4	196	2	149	4	232	3	174	ND	
XP_001706797	19705	Ribosomal protein L11	4	219	5	332	3	166	3	179	ND	

			Trophozoites								Cyst	
**Encystation induction time**			**0 hours**		**6 hours**		**12 hours**		**24 hours**		**48 hours**	
**Accession number**	**Mass**	**Description**	Peptides	Mascot score	Peptides	Mascot score	Peptides	Mascot score	Peptides	Mascot score	Peptides	Mascot score
XP_001704829	22839	Ribosomal protein L13a	9	533	9	564	7	384	7	488	3	232
XP_001709584	14732	Ribosomal protein L14	ND		2	133	ND		ND		ND	
XP_001707606	18650	Ribosomal protein L17	ND		ND		ND		2	68	ND	
XP_001709691	20130	Ribosomal protein L18	2	116	4	174	2	130	3	173	2	119
XP_001705001	20552	Ribosomal protein L18a	ND		2	73	ND		ND		ND	
XP_001710022	18227	Ribosomal protein L21	6	284	7	432	3	104	5	225	ND	
XP_001706142	15994	Ribosomal protein L23A	5	334	4	371	ND		ND		ND	
XP_001705268	11950	Ribosomal protein L24A	2	112	2	95	ND		ND		ND	
XP_001709840	15322	Ribosomal protein L27	7	578	6	539	6	459	7	605	4	313
XP_001704050	17100	Ribosomal protein L27a	ND		2	135	ND		3	157	ND	
XP_001709269	11659	Ribosomal protein L30	2	94	2	103	2	116	ND		ND	
XP_001706140	12132	Ribosomal protein L31B	4	244	4	311	4	247	3	207	ND	
XP_001706078	15919	Ribosomal protein L32	2	150	4	232	3	174	3	219	2	149
XP_001706089	13471	Ribosomal protein L34	2	110	4	258	ND		ND		ND	
XP_001706548	13862	Ribosomal protein L35a	8	518	ND		5	296	3	297	2	134
XP_001704191	10534	Ribosomal protein L36-1	ND		ND		2	73	ND		ND	
XP_001706189	10276	Ribosomal protein L37	ND		3	179	2	141	4	190	ND	
XP_001705992	26688	Ribosomal protein S2	5	386	3	172	2	143	ND		3	153
XP_001708724	24703	Ribosomal protein S3	2	212	8	466	5	388	4	312	ND	
XP_001705170	28327	Ribosomal protein S3a	2	286	5	428	3	213	ND		2	137
XP_001707248	30374	Ribosomal protein S4	6	274	7	408	4	217	ND		3	167
XP_001704024	20993	Ribosomal protein S5	ND		2	131	2	101	ND		ND	
XP_001706339	21621	Ribosomal protein S9	ND		2	138	ND		ND		2	65
XP_001705221	15355	Ribosomal protein S10B	8	370	2	103	8	364	6	285	ND	
EES99335	21890	Ribosomal protein S11	10	541	7	518	2	84	6	392	ND	
XP_001704776	14326	Ribosomal protein S12	6	345	ND		4	226	5	273	ND	
XP_001706152	15698	Ribosomal protein S14	7	507	2	137	5	426	4	291	ND	
XP_001706502	17569	Ribosomal protein S16	3	227	3	210	ND		ND		ND	
XP_001709586	14024	Ribosomal protein S20	2	138	2	100	3	128	2	97	ND	
XP_001705391	12735	Ribosomal protein S26	2	83	ND		ND		ND		ND	
XP_001708809	15442	Ribosomal protein S29A	5	247	ND		ND		ND		ND	
**Folding**												
XP_001704733	67330	Chaperone protein dnaJ	5	297	2	97	5	242	5	348	2	233
XP_001705621	94719	Heat-shock protein, putative	7	522	3	174	ND		2	169	ND	
BAD83616	39977	cytosolic-type hsp90 N-Terminal (HSP90-alpha GI:157436100)	4	175	ND		ND		ND		ND	
BAD83617	40711	cytosolic-type hsp90 C-Terminal (HSP90-alpha GI:157433563)	3	155	ND		ND		ND		ND	
XP_001709001	97685	Caseinolityc peptidase B ClpB (chaperone ATPase)	7	323	ND		ND		ND		ND	
XP_001706090	25742	Protein disulfide isomerase PDI1	2	131	ND		2	69	17	1370	14	1131
XP_001707782	50357	Protein disulfide isomerase PDI2	2	213	ND		5	265	ND		2	180

			Trophozoites								Cyst	
**Encystation induction time**			**0 hours**		**6 hours**		**12 hours**		**24 hours**		**48 hours**	
**Accession number**	**Mass**	**Description**	Peptides	Mascot score	Peptides	Mascot score	Peptides	Mascot score	Peptides	Mascot score	Peptides	Mascot score
**Transport, sorting and endocytosis**												
XP_001704962	79444	Dynamin	14	1050	18	1237	2	172	6	396	3	169
AAM83403	206832	putative clathrin heavy chain	9	548	3	137	14	827	5	276	ND	
XP_001708133	86961	Alpha adaptin (AP-2 alpha)	2	178	ND		ND		ND		ND	
AAM27210	122887	putative adaptor protein complex large chain subunit BetaB (AP-2 Beta)	4	252	ND		3	174	ND		ND	
XP_001709302	87429	Vacuolar protein sorting 35 (RETROMER endosomes to golgi)	2	105	7	347	ND		ND		ND	
XP_001704135	71473	Vacuolar ATP synthase catalytic subunit A	3	259	ND		ND		9	622	ND	
XP_001705680	54694	Vacuolar ATP synthase subunit B	ND		2	136	ND		ND		ND	
XP_001709085	467183	Lipopolysaccharide-responsive and beige-like anchor protein	2	164	5	315	ND		ND		ND	
XP_001706385	26310	Synaptobrevin-like protein	ND		2	69	ND		ND		ND	
XP_001703953	90584	NSF (N-ethylmaleimide sensitive factor - ATPase)	8	539	ND		ND		ND		ND	
XP_001704291	89529	NSF (N-ethylmaleimide sensitive factor - ATPase)	2	419	ND		ND		ND		ND	
XP_001709737	114526	Kinesin-3	16	1223	2	115	2	72	ND		ND	
XP_001709752	120423	Kinesin-3	3	929	ND		ND		ND		ND	
**Cytoskeleton**												
XP_001704310	33849	Alpha-1 giardin	3	174	ND		ND		ND		11	595
XP_001708837	33280	Alpha-3 giardin	ND		ND		ND		ND		6	398
XP_001705104	37311	Alpha-10 giardin	4	217	ND		ND		ND		ND	
AAN78305	48858	alpha-tubulin	2	194	ND		2	292	ND		5	417
CAA29923	49777	beta-tubulin	2	91	2	338	ND		2	355	3	350
XP_001709528	88582	VSP	ND		4	746	ND		ND		ND	
XP_001708046	25847	VSP	ND		ND		ND		ND		2	168
XP_001704987	73979	Flagella associated protein	ND		ND		ND		ND		2	71
XP_001706983	76693	VSP with INR Variant-specific surface protein (WB-9B10)	ND		ND		ND		2	145	6	304
**Proteases**												
XP_001710286	78575	Dipeptidyl-peptidase III	6	302	ND		ND		ND		2	89
XP_001706477	49622	Xaa-Pro dipeptidase	2	89	ND		ND		ND		ND	
XP_001704423	61799	Cathepsin L precursor	2	146	ND		ND		ND		ND	
XP_001704875	128408	Metalloprotease, insulinase family	ND		ND		ND		2	81	ND	
**Ubiquitin-Proteasome system**												
CAA49657	8597	ubiquitin	3	386	2	212	2	225	3	232	ND	
XP_001708030	121326	Ubiquitin-conjugating enzyme E1	17	1185	5	342	ND		ND		ND	
XP_001705864	20249	Ubiquitin-conjugating enzyme E2-17 kDa 3	2	76	ND		ND		ND		ND	
XP_001706028	135785	26S proteasome non-ATPase regulatory subunit 2 (Rpn1)	ND		2	112	ND		ND		ND	
XP_001709153	147519	26S proteasome regulatory subunit, putative (Rpn2)	4	236	ND		ND		ND		ND	
**N-acetylgalactosamine synthesis (GalNAc)**												
XP_001709043	29385	Glucosamine-6-phosphate isomerase GNP	ND		ND		ND		9	565	4	234
XP_001708988	22784	Glucose 6-phosphate N-acetyltransferase GNA	ND		ND		ND		3	263	ND	
AAO31976	56389	phosphoacetylglucosamine mutase AGM	ND		ND		ND		4	301	ND	

			Trophozoites								Cyst	
**Encystation induction time**			**0 hours**		**6 hours**		**12 hours**		**24 hours**		**48 hours**	
**Accession number**	**Mass**	**Description**	Peptides	Mascot score	Peptides	Mascot score	Peptides	Mascot score	Peptides	Mascot score	Peptides	Mascot score
**Metabolic enzymes**												
XP_001705744	78033	Acetyl-CoA synthetase	11	759	3	270	13	813	3	205	ND	
XP_001710279	106638	Acyl-CoA synthetase	3	190	ND		ND		7	434	2	89
XP_001704525	58268	Alanine aminotransferase, putative	4	225	ND		ND		2	83	ND	
XP_001710238	97053	Alcohol dehydrogenase	ND		15	1022	ND		ND		17	1037
XP_001705763	44777	Alcohol dehydrogenase lateral transfer candidate	ND		ND		2	98	ND		ND	
XP_001705755	64072	Arginine deiminase	25	1496	2	88	12	890	13	909	ND	
XP_001707670	46574	A-type flavoprotein lateral transfer candidate	9	459	9	517	ND		4	241	ND	
XP_001704865	91419	1,4-alpha-glucan branching enzyme	5	287	ND		ND		ND		ND	
XP_001705733	195727	4-alpha-glucanotransferase, amylo-alpha-1,6-glucosidase	ND		ND		ND		ND		11	785
XP_001706738	65031	2,3-bisphosphoglycerate-independent phosphoglycerate mutase	ND		ND		ND		18	1330	ND	
XP_001709668	67960	CTP synthase	2	134	ND		ND		ND		ND	
EFF74053	31682	dihydrodipicolinate synthase	ND		ND		ND		ND		2	100
XP_001705909	63783	DRAP deaminase	3	213	ND		4	280	2	160	ND	
XP_001709477	46183	Farnesyl diphosphate synthase	4	224	ND		ND		ND		ND	
XP_001710050	35173	Fructose-bisphosphate aldolase	25	1844	ND		30	1879	23	1492	ND	
XP_001706734	37670	Glucokinase	3	192	ND		ND		ND		ND	
XP_001705324	98240	Glutamate synthase	4	301	ND		2	125	8	566	ND	
AAB18421	36280	glyceraldehyde 3-phosphate dehydrogenase	13	976	10	721	11	730	5	303	2	97
XP_001706726	84990	Glycogen synthase, putative	4	277	ND		2	89	4	251	ND	
XP_001709411	84475	Long chain fatty acid CoA ligase, putative	5	291	ND		ND		ND		ND	
XP_001707118	35453	Malate dehydrogenase	2	183	ND		ND		ND		ND	
AAL59603	51439	NADH oxidase	ND		8	480	ND		ND		ND	
XP_001707974	53283	NADH oxidase lateral transfer candidate	11	665	16	1196	ND		20	1461	26	1979
XP_001706095	16764	Nucleoside diphosphate kinase	ND		2	177	ND		ND		ND	
AAG47713	72005	phosphoenolpyruvate carboxykinase	ND		4	264	ND		ND		3	151
XP_001706998	43555	Phosphoglycerate kinase	5	322	7	583	9	687	6	471	ND	
XP_001705528	39272	Phosphatidylinositol transfer protein alpha isoform	ND		2	74	ND		ND		ND	
XP_001708591	88378	Purine nucleoside phosphorylase lateral transfer candidate	3	150	ND		ND		ND		5	307
XP_001707507	59907	Pyrophosphate-fructose 6-phosphate 1-phosphotransferase alpha subunit	14	979	ND		ND		5	182	ND	
XP_001708948	131700	Pyruvate-flavodoxin oxidoreductase	4	222	ND		22	1192	53	3327	26	
XP_001708704	138821	Pyruvate-flavodoxin oxidoreductase	2	114	23	1293	ND		4	206	4	191
AAD55757	56004	putative pyruvate:ferredoxin oxidoreductase 2	2	88	2	89	ND		ND		5	253
XP_001709529	60468	Pyruvate kinase	16	1311	3	127	17	1330	13	837	ND	
AAF19604	57352	pyruvate kinase	5	306	ND		2	157	3	163	ND	
AAC47168	97563	pyruvate,phosphate dikinase	2	195	36	2477	12	730	21	1442	6	442
AAD51093.1	22464	thioredoxin peroxidase homolog	5	259	ND		ND		ND		ND	
AAM94634	63181	threonine dehydratase	4	242	ND		ND		ND		ND	
AAB01342	27837	triose phosphate isomerase	3	158	ND		ND		ND		ND	
XP_001707342	34243	UPL-1 Uridine phosporylase	7	372	2	138,68	ND		ND		ND	

			Trophozoites								Cyst	
**Encystation induction time**			**0 hours**		**6 hours**		**12 hours**		**24 hours**		**48 hours**	
**Accession number**	**Mass**	**Description**	Peptides	Mascot score	Peptides	Mascot score	Peptides	Mascot score	Peptides	Mascot score	Peptides	Mascot score
XP_001704181	65984	Uridine kinase	3	182	ND		ND		ND		ND	
**Kinases and phosphatases (signalling)**												
XP_001704895	76275	Kinase	ND		4	259	4	251	3	202	ND	
XP_001709026	133112	Kinase, NEK	7	482	ND		ND		ND		ND	
XP_001707221	124703	Kinase, NEK	ND		ND		ND		ND		4	175
XP_001707357	90500	Kinase, NEK	12	648	7	470	3	242	2	78	ND	
XP_001704268	66922	Kinase, NEK	ND		4	212	ND		ND		ND	
XP_001708517	53003	Kinase, NEK	ND		2	157	ND		ND		ND	
XP_001706081	41498	Kinase, NEK	2	120	ND		ND		ND		ND	
XP_001709229	34298	Kinase, NEK	ND		ND		2	95	ND		ND	
XP_001708873	33835	Kinase, NEK	2	110	ND		ND		ND		ND	
XP_001710036	32597	Kinase, NEK	3	122	9	578	4	267	ND		3	128
XP_001709632	112591	Kinase, NEK-frag	3	135	ND		ND		ND		ND	
XP_001705714	189834	Kinase, CDC7	10	611	ND		3	131	5	284	ND	
XP_001705269	46050	Kinase, CAMK CAMKL	2	79	4	223	ND		ND		ND	
XP_001704849	97169	Kinase, CMGC DYRK	2	202	ND		ND		ND		2	149
XP_001706920	54793	Kinase, CMGC CK2	4	239	ND		ND		ND		ND	
XP_001709931	32823	Kinase, CMGC CDK	3	119	ND		ND		ND		ND	
XP_001704058	35197	Kinase, CMGC CDK	2	103	ND		ND		ND		ND	
XP_001705776	77123	Kinase, PLK	2	111	ND		ND		ND		ND	
XP_001707209	103239	Kinase, SCY1	2	116	ND		ND		ND		ND	
XP_001705743	72237	Phosphatase, putative	3	160	2	89	3	123	2	89	ND	
XP_001707645	39732	Ser/Thr phosphatase PP2A-2 catalytic subunit	2	109	ND		ND		ND		ND	
XP_001705519	41253	Developmentally regulated GTP-binding protein 1	ND		ND		2	164	ND		ND	
XP_001708553	51320	G1 to S phase transition protein 1, putative	ND		ND		2	79	ND		ND	
AAN73430	41232	extracellular signal-regulated kinase 2	ND		4	333	ND		ND		ND	
**Protein 21.1 (Ankirin domains involved in protein-protein interaction)**												
XP_001710268	125741	Protein 21.1	3	195	2	178	2	123	ND		2	75
XP_001710302	124254	Protein 21.1	6	387	ND		6	469	ND		3	241
XP_001705792	113860	Protein 21.1	3	126	ND		ND		ND		ND	
XP_001709256	112759	Protein 21.1	ND		3	143	ND		ND		ND	
XP_001705064	95163	Protein 21.1	18	1463	3	201	4	294	3	176	ND	
XP_001705461	93867	Protein 21.1	2	124	ND		ND		ND		ND	
XP_001709481	87536	Protein 21.1	ND		ND		ND		ND		2	131
XP_001708876	79617	Protein 21.1	ND		ND		ND		ND		5	431
XP_001704344	64147	Protein 21.1	7	369	ND		2	57	ND		ND	
XP_001704817	59732	Protein 21.1	ND		ND		ND		ND		4	187
XP_001708396	36947	Protein 21.1	ND		2	133	ND		ND		3	160
XP_001704622	25568	Protein 21.1	ND		ND		ND		ND		2	150

			Trophozoites								Cyst	
**Encystation induction time**			**0 hours**		**6 hours**		**12 hours**		**24 hours**		**48 hours**	
**Accession number**	**Mass**	**Description**	Peptides	Mascot score	Peptides	Mascot score	Peptides	Mascot score	Peptides	Mascot score	Peptides	Mascot score
**Hypothetical proteins**												
XP_001705438	80094	Hypothetical protein GL50803_115478	ND		2	107	ND		ND		3	188
XP_001707402	149661	Hypothetical protein GL50803_101278	4	262	ND		5	310	3	147	ND	
XP_001708344	53337	Hypothetical protein GL50803_94463	ND		3	141	ND		ND		4	227
XP_001706533	240913	Hypothetical protein GL50803_94117	5	266	2	146	9	490	8	416	ND	
XP_001706938	26704	Hypothetical protein GL50803_28699	2	124	ND		ND		ND		ND	
XP_001707013	15445	Hypothetical protein GL50803_24451	ND		ND		ND		ND		3	119
XP_001707206	49034	Hypothetical protein GL50803_23017	2	116	ND		ND		ND		2	67
XP_001707920	146544	Hypothetical protein GL50803_22543	2	61	ND		ND		ND		ND	
XP_001706898	42334	Hypothetical protein GL50803_21628	2	107	ND		ND		ND		ND	
XP_001708629	35393	Hypothetical protein GL50803_17400	2	70	ND		ND		ND		ND	
XP_001706499	179239	Hypothetical protein GL50803_17332	23	1436	ND		ND		ND		ND	
XP_001704754	26971	Hypothetical protein GL50803_16794	2	164	ND		ND		ND		ND	
XP_001707556	103032	Hypothetical protein GL50803_16653	ND		ND		ND		2	82	ND	
XP_001707576	88291	Hypothetical protein GL50803_16648	ND		ND		ND		ND		2	147
XP_001707037	29665	Hypothetical protein GL50803_16424	ND		ND		ND		ND		2	110
XP_001709409	83582	Hypothetical protein GL50803_16353	5	313	4	251	2	139	2	122	ND	
XP_001704054	110474	Hypothetical protein GL50803_16312	2	148	ND		ND		ND		ND	
XP_001707019	52068	Hypothetical protein GL50803_11380	3	173	ND		6	360	ND		ND	
XP_001706436	42667	Hypothetical protein GL50803_9861	5	483	4	220	4	240	2	137	ND	
XP_001707432	213593	Hypothetical protein GL50803_9183	ND		ND		ND		24	1472	ND	
XP_001708788	52292	Hypothetical protein GL50803_9098	3	149	ND		ND		ND		ND	
XP_001709369	28631	Hypothetical protein GL50803_8770	3	143	ND		2	111	ND		ND	
XP_001704451	37155	Hypothetical protein GL50803_8692	3	226	4	233	ND		ND		ND	
XP_001706561	56796	Hypothetical protein GL50803_8528	15	804	ND		12	690	7	457	ND	
XP_001709157	21146	Hypothetical protein GL50803_7244	ND		2	81	ND		ND		ND	
XP_001704720	63159	Hypothetical protein GL50803_6617	3	164	ND		ND		ND		ND	
XP_001705314	17331	Hypothetical protein GL50803_6171	2	157	6	382	2	97	ND		ND	
XP_001707179	29613	Hypothetical protein GL50803_5800	2	138	ND		ND		ND		ND	
XP_001704815	170142	Hypothetical protein GL50803_4595	3	196	ND		ND		4	162	ND	
XP_001705344.1	72703	Hypothetical protein GLP15_1799	3	205	ND		ND		ND		ND	
**Others**												
XP_001704350	41094	HZGJ (WD40 Domain)	2	106	ND		ND		ND		ND	
XP_001709096	38488	WD-40 repeat protein family	ND		3	101	ND		ND		ND	
XP_001706755	28540	14-3-3 protein	ND		2	112	ND		ND		ND	
**TOTAL**			**151**		**95**		**70**		**67**		**57**	

ND: Not detected
