# Supplementary material for: Ubiquitination dynamics in the early-branching eukaryote Giardia intestinalis
Source: Microbiologyopen. 2013 Apr 23;2(3):525–39. doi: 10.1002/mbo3.88 (PMC3684764; doi:10.1002/mbo3.88)
Supplement: Supplementary file 5 [file mbo30002-0525-SD5.pdf]

Supplementary Table 2
Proteins presents in both control and *in-vitro* ubiquitination assay

Trophozoites (0 hours of induction)				
**Accession number**	**Mass**	**Description**	Peptides	Mascot score
XP_001707321	45169	Elongation factor 1-gamma	3	184
XP_001705202	49461	Acid sphingomyelinase-like phosphodiesterase 3b precursor	11	753
XP_001704525	58268	Alanine aminotransferase, putative	11	583
XP_001710238	97053	Alcohol dehydrogenase	12	740
XP_001705763	44777	Alcohol dehydrogenase lateral transfer candidate	8	595
XP_001704310	33849	Alpha-1 giardin	3	149
XP_001704865	91419	1,4-alpha-glucan branching enzyme	11	573
XP_001709888	137989	4-alpha-glucanotransferase	4	349
XP_001705176	55050	Aminoacyl-histidine dipeptidase	4	167
XP_001705755	64072	Arginine deiminase	10	693
XP_001706738	65031	2,3-bisphosphoglycerate-independent phosphoglycerate mutase	18	1275
2ISV_A	35203	Chain A, Structure Of Giardia Fructose-1,6-Biphosphate Aldolase In Complex With Phosphoglycolohydroxamate	15	678
XP_001708626	40764	Chaperone protein dnaJ	8	625
XP_001705909	63783	DRAP deaminase	2	103
XP_001704962	79444	Dynamin	14	864
XP_001706428	10423	Dynein light chain	6	353
AAN73430	41232	extracellular signal-regulated kinase 2	5	356
XP_001706925	23825	FKBP-type peptidyl-prolyl cis-trans isomerase	2	126
XP_001710050	35173	Fructose-bisphosphate aldolase	18	1133
XP_001706734	37670	Glucokinase	5	241
XP_001709043	29385	Glucosamine-6-phosphate deaminase	5	378
XP_001704441	83004	Glucose-6-phosphate 1-dehydrogenase	15	1125
XP_001708857	64433	Glucose-6-phosphate isomerase	21	1339
XP_001705324	98240	Glutamate synthase	3	151
AAB18421	36280	glyceraldehyde 3-phosphate dehydrogenase	5	344
XP_001707988	118760	Glycerol-3-phosphate dehydrogenase	18	1370
XP_001705977	104765	Glycogen phosphorylase	43	2968
XP_001705621	94719	Heat-shock protein, putative	13	667
XP_001704350	41094	HZGJ	2	268
XP_001704895	76275	Kinase	10	531
XP_001708517	53003	Kinase, NEK	5	392
XP_001708873	33835	Kinase, NEK	2	126
XP_001705891	94310	Long chain fatty acid CoA ligase 5	7	336
XP_001707118	35453	Malate dehydrogenase	10	569
AAC47396	61459	malic enzyme	23	1570
XP_001708073	34656	Metallo-beta-lactamase superfamily protein	6	377
XP_001709257	46955	NADH oxidase	12	694
AAL59603	51439	NADH oxidase	12	694
P28724	49715	RecName: Full=NADP-specific glutamate dehydrogenase	10	1064
XP_001706095	16764	Nucleoside diphosphate kinase	4	258
XP_001709979	36394	Ornithine carbamoyltransferase	22	1187
XP_001707244	49507	Ornithine decarboxylase	6	265
CAC33857	21920	ORF-C4	14	944
XP_001704832	27620	Peroxiredoxin 1	5	295
XP_001705094	23170	20S proteasome alpha subunit 4	7	419
XP_001706090	25742	Protein disulfide isomerase PDI1	12	807
XP_001705792	113860	Protein 21.1	7	476
XP_001709256	112759	Protein 21.1	2	146
XP_001705461	93867	Protein 21.1	7	619
XP_001709481	87536	Protein 21.1	5	372
XP_001708927	66176	Protein 21.1	13	880
XP_001704344	64147	Protein 21.1	22	1502
XP_001704817	59732	Protein 21.1	4	305
XP_001708948	131700	Pyruvate-flavodoxin oxidoreductase	3	204
AAC47168	97563	pyruvate,phosphate dikinase	7	555
XP_001709529	60468	Pyruvate kinase	6	407
XP_001707507	59907	Pyrophosphate-fructose 6-phosphate 1-phosphotransferase alpha subunit	16	992

Accession number	Mass	Description	Peptides	Mascot score
XP_001705797	20823	Ribosomal protein L9	3	207
XP_001704829	22839	Ribosomal protein L13a	5	237
XP_001705001	20552	Ribosomal protein L18a	5	258
XP_001706089	13471	Ribosomal protein L34	2	145
XP_001706548	13862	Ribosomal protein L35a	3	191
XP_001705221	15355	Ribosomal protein S10B	4	200
XP_001706152	15698	Ribosomal protein S14	4	265
XP_001705652	14721	Ribosomal protein S15A	4	168
XP_001704088	17443	Ribosomal protein S18	7	443
XP_001705100	78963	Threonyl-tRNA synthetase	10	593
XP_001707324	103951	Threonine dehydratase lateral transfer candidate	10	633
XP_001704614	77497	Transketolase	28	1819
XP_001706747	12110	Translation initiation factor	2	111
XP_001704069	24724	Translation elongation factor	3	264
CAA49657	8597	ubiquitin	2	124
XP_001707342	34243	UPL-1	3	130
XP_001704852	89521	VSP	4	261
XP_001706983	76693	VSP with INR	9	565
XP_001707418	80704	Hypothetical protein GL50803_137685	3	111
XP_001704133	163092	Hypothetical protein GL50803_137753	4	295
XP_001708895	24005	Hypothetical protein GL50803_135885	7	528
XP_001707402	149661	Hypothetical protein GL50803_101278	24	1252
XP_001706533	240913	Hypothetical protein GL50803_94117	3	106
XP_001704399	89458	Hypothetical protein GL50803_24279	2	130
XP_001706499	179239	Hypothetical protein GL50803_17332	5	321
XP_001707081	35170	Hypothetical protein GL50803_16067	3	202
XP_001708469	38187	Hypothetical protein GL50803_15581	8	544
XP_001707432	213593	Hypothetical protein GL50803_9183	57	3804
XP_001705178	47560	Hypothetical protein GL50803_8405	3	194
XP_001709157	21146	Hypothetical protein GL50803_7244	3	139
XP_001704651	141975	Hypothetical protein GL50803_7130	5	329

6 hours of induction

Accession number	Mass	Description	Peptides	Mascot score
XP_001710279	106638	Acyl-CoA synthetase	3	186
XP_001710238	97053	Alcohol dehydrogenase	23	1480
XP_001705763	44777	Alcohol dehydrogenase lateral transfer candidate	2	227
XP_001704865	91419	1,4-alpha-glucan branching enzyme	5	303
XP_001705176	55050	Aminoacyl-histidine dipeptidase	4	176
XP_001705755	64072	Arginine deiminase	2	123
XP_001706738	65031	2,3-bisphosphoglycerate-independent phosphoglycerate mutase	15	955
2ISV_A	35203	Chain A, Structure Of Giardia Fructose-1,6-Biphosphate Aldolase In Complex With Phosphoglycolohydroxamate	5	339
XP_001708626	40764	Chaperone protein dnaJ	2	67
XP_001706428	10423	Dynein light chain	4	271
XP_001708857	64433	Glucose-6-phosphate isomerase	16	936
XP_001705007	87528	Glutaminyl-tRNA synthetase	3	157
XP_001707988	118760	Glycerol-3-phosphate dehydrogenase	7	377
XP_001705977	104765	Glycogen phosphorylase	43	2668
XP_001706726	84990	Glycogen synthase, putative	7	583
XP_001705621	94719	Heat-shock protein, putative	6	363
XP_001707118	35453	Malate dehydrogenase	3	173
AAC47396	61459	malic enzyme	3	180
AAL59603	51439	NADH oxidase	13	852
XP_001706095	16764	Nucleoside diphosphate kinase	6	409
XP_001709979	36394	Ornithine carbamoyltransferase	12	650
XP_001707244	49507	Ornithine decarboxylase	4	230
CAC33857	21920	ORF-C4	10	694
XP_001706090	25742	Protein disulfide isomerase PDI1	2	182
XP_001705064	95163	Protein 21.1	23	2114
XP_001708927	66176	Protein 21.1	8	493

Accession number	Mass	Description	Peptides	Mascot score
XP_001704344	64147	Protein 21.1	4	231
XP_001708948	131700	Pyruvate-flavodoxin oxidoreductase	16	1041
AAC47168	97563	pyruvate,phosphate dikinase	3	200
XP_001707507	59907	Pyrophosphate-fructose 6-phosphate 1-phosphotransferase alpha subunit	2	127
XP_001706321	25348	Ribosomal protein L7a	2	100
XP_001705797	20823	Ribosomal protein L9	3	231
XP_001704829	22839	Ribosomal protein L13a	7	321
XP_001705001	20552	Ribosomal protein L18a	5	284
XP_001708724	24703	Ribosomal protein S3	2	144
XP_001705221	15355	Ribosomal protein S10B	3	182
XP_001706152	15698	Ribosomal protein S14	2	196
XP_001705652	14721	Ribosomal protein S15A	6	335
XP_001704088	17443	Ribosomal protein S18	12	646
XP_001708809	15442	Ribosomal protein S29A	2	130
XP_001705100	78963	Threonyl-tRNA synthetase	16	1040
AAM94634	63181	threonine dehydratase	2	118
XP_001704614	77497	Transketolase	6	294
XP_001704069	24724	Translation elongation factor	2	237
XP_001704135	71473	Vacuolar ATP synthase catalytic subunit A	2	165
XP_001706983	76693	VSP with INR	10	543
XP_001707402	149661	Hypothetical protein GL50803_101278	4	148
XP_001707432	213593	Hypothetical protein GL50803_9183	23	1609
XP_001709157	21146	Hypothetical protein GL50803_7244	4	196
XP_001706349	12010	Hypothetical protein GL50803_5890	3	172

12 hours of induction

Accession number	Mass	Description	Peptides	Mascot score
XP_001705202	49461	Acid sphingomyelinase-like phosphodiesterase 3b precursor	10	710
XP_001704525	58268	Alanine aminotransferase, putative	5	192
XP_001710238	97053	Alcohol dehydrogenase	19	1143
XP_001705763	44777	Alcohol dehydrogenase lateral transfer candidate	6	435
XP_001705176	55050	Aminoacyl-histidine dipeptidase	14	820
XP_001705755	64072	Arginine deiminase	3	139
XP_001706738	65031	2,3-bisphosphoglycerate-independent phosphoglycerate mutase	22	1517
XP_001709001	97685	ClpB protein	5	199
2ISV_A	35203	Chain A, Structure Of Giardia Fructose-1,6-Biphosphate Aldolase In Complex With Phosphoglycolohydroxamate	23	1317
XP_001710286	78575	Dipeptidyl-peptidase III	2	135
XP_001706428	10423	Dynein light chain	5	346
AAN73430	41232	extracellular signal-regulated kinase 2	4	319
XP_001709477	46183	Farnesyl diphosphate synthase	3	185
XP_001706734	37670	Glucokinase	11	613
XP_001709043	29385	Glucosamine-6-phosphate deaminase	18	1074
XP_001704441	83004	Glucose-6-phosphate 1-dehydrogenase	12	832
XP_001708857	64433	Glucose-6-phosphate isomerase	33	2171
AAB18421	36280	glyceraldehyde 3-phosphate dehydrogenase	3	207
XP_001707988	118760	Glycerol-3-phosphate dehydrogenase	16	1251
XP_001705977	104765	Glycogen phosphorylase	41	2950
XP_001705621	94719	Heat-shock protein, putative	7	415
XP_001704350	41094	HZGJ	3	284
XP_001704895	76275	Kinase	8	494
XP_001706267	71458	Kinase, NEK	2	116
XP_001704268	66922	Kinase, NEK	9	481
XP_001708517	53003	Kinase, NEK	7	523
XP_001708873	33835	Kinase, NEK	3	157
XP_001705776	77123	Kinase, PLK	3	120
XP_001709085	467183	Lipopolysaccharide-responsive and beige-like anchor protein	2	76
XP_001707118	35453	Malate dehydrogenase	7	522
AAC47396	61459	malic enzyme	30	2111
XP_001708073	34656	Metallo-beta-lactamase superfamily protein	10	594
XP_001709257	46955	NADH oxidase	17	1155

Accession number	Mass	Description	Peptides	Mascot score
AAL59603	51439	NADH oxidase	8	517
P28724	49715	RecName: Full=NADP-specific glutamate dehydrogenase	21	1836
XP_001706095	16764	Nucleoside diphosphate kinase	6	413
XP_001709979	36394	Ornithine carbamoyltransferase	24	1430
XP_001707244	49507	Ornithine decarboxylase	10	527
CAC33857	21920	ORF-C4	18	1046
XP_001705094	23170	20S proteasome alpha subunit 4	5	321
XP_001706028	135785	26S proteasome non-ATPase regulatory subunit 2	21	1294
XP_001706090	25742	Protein disulfide isomerase PDI1	10	785
XP_001709256	112759	Protein 21.1	5	254
XP_001705461	93867	Protein 21.1	5	449
XP_001709481	87536	Protein 21.1	8	489
XP_001708927	66176	Protein 21.1	11	765
XP_001704344	64147	Protein 21.1	26	1680
XP_001704817	59732	Protein 21.1	8	511
XP_001709808	57078	Protein 21.1	4	218
XP_001708591	88378	Purine nucleoside phosphorylase lateral transfer candidate	3	141
AAF19604	57352	pyruvate kinase	12	800
XP_001707507	59907	Pyrophosphate-fructose 6-phosphate 1-phosphotransferase alpha subunit	8	523
XP_001705231	27023	Ribosomal protein L7	2	72
XP_001705797	20823	Ribosomal protein L9	2	137
XP_001705001	20552	Ribosomal protein L18a	5	314
XP_001705221	15355	Ribosomal protein S10B	4	214
XP_001706152	15698	Ribosomal protein S14	5	276
XP_001704088	17443	Ribosomal protein S18	5	337
XP_001705100	78963	Threonyl-tRNA synthetase	20	1210
XP_001707324	103951	Threonine dehydratase lateral transfer candidate	6	345
XP_001704614	77497	Transketolase	23	1418
XP_001704069	24724	Translation elongation factor	3	255
XP_001709096	38488	WD-40 repeat protein family	2	102
XP_001704133	163092	Hypothetical protein GL50803_137753	4	255
XP_001708895	24005	Hypothetical protein GL50803_135885	9	585
XP_001707402	149661	Hypothetical protein GL50803_101278	15	1039
XP_001706533	240913	Hypothetical protein GL50803_94117	6	344
XP_001704399	89458	Hypothetical protein GL50803_24279	13	777
XP_001709409	83582	Hypothetical protein GL50803_16353	2	96
XP_001708469	38187	Hypothetical protein GL50803_15581	7	487
XP_001707432	213593	Hypothetical protein GL50803_9183	89	6400
XP_001704451	37155	Hypothetical protein GL50803_8692	2	84
XP_001705178	47560	Hypothetical protein GL50803_8405	3	195
XP_001709157	21146	Hypothetical protein GL50803_7244	3	142
XP_001704720	63159	Hypothetical protein GL50803_6617	2	117

24 hours of induction

Accession number	Mass	Description	Peptides	Mascot score
XP_001710238	97053	Alcohol dehydrogenase	14	912
XP_001706738	65031	2,3-bisphosphoglycerate-independent phosphoglycerate mutase	3	233
2ISV_A	35203	Chain A, Structure Of Giardia Fructose-1,6-Biphosphate Aldolase In Complex With Phosphoglycolohydroxamate	2	172
XP_001706428	10423	Dynein light chain	4	314
XP_001709043	29385	Glucosamine-6-phosphate deaminase	19	1306
XP_001708857	64433	Glucose-6-phosphate isomerase	4	294
XP_001705977	104765	Glycogen phosphorylase	20	1411
XP_001704268	66922	Kinase, NEK	5	297
XP_001710036	32597	Kinase, NEK	2	64
XP_001707118	35453	Malate dehydrogenase	2	173
AAC47396	61459	malic enzyme	16	1109
AAL59603	51439	NADH oxidase	18	1285
P28724	49715	RecName: Full=NADP-specific glutamate dehydrogenase	17	1092
XP_001706095	16764	Nucleoside diphosphate kinase	2	98
XP_001709979	36394	Ornithine carbamoyltransferase	9	639

Accession number	Mass	Description	Peptides	Mascot score
CAC33857	21920	ORF-C4	11	815
XP_001705094	23170	20S proteasome alpha subunit 4	3	145
XP_001706090	25742	Protein disulfide isomerase PDI1	8	575
XP_001710302	124254	Protein 21.1	3	161
XP_001708927	66176	Protein 21.1	8	618
XP_001704344	64147	Protein 21.1	7	379
XP_001709808	57078	Protein 21.1	3	209
XP_001708948	131700	Pyruvate-flavodoxin oxidoreductase	15	912
XP_001707507	59907	Pyrophosphate-fructose 6-phosphate 1-phosphotransferase alpha subunit	14	947
XP_001704614	77497	Transketolase	2	72
XP_001704069	24724	Translation elongation factor	2	124
XP_001707432	213593	Hypothetical protein GL50803_9183	15	929

48 hours of induction

Accession number	Mass	Description	Peptid	Mascot score
XP_001705202	49461	Acid sphingomyelinase-like phosphodiesterase 3b precursor	3	206
XP_001710238	97053	Alcohol dehydrogenase	21	1279
XP_001704310	33849	Alpha-1 giardin	4	225
XP_001704865	91419	1,4-alpha-glucan branching enzyme	2	94
XP_001705733	195727	4-alpha-glucanotransferase, amylo-alpha-1,6-glucosidase	3	250
AAN78305	48858	alpha-tubulin	2	157
CAA29923	49777	beta-tubulin	3	177
XP_001706738	65031	2,3-bisphosphoglycerate-independent phosphoglycerate mutase	16	1012
2ISV_A	35203	Chain A, Structure Of Giardia Fructose-1,6-Biphosphate Aldolase In Complex With Phosphoglycolohydroxamate	13	769
XP_001708626	40764	Chaperone protein dnaJ	6	345
XP_001704962	79444	Dynamin	4	226
XP_001706428	10423	Dynein light chain	4	297
XP_001706734	37670	Glucokinase	5	317
XP_001709043	29385	Glucosamine-6-phosphate deaminase	20	1395
XP_001704441	83004	Glucose-6-phosphate 1-dehydrogenase	8	551
XP_001708857	64433	Glucose-6-phosphate isomerase	32	2131
XP_001705007	87528	Glutaminyl-tRNA synthetase	4	231
AAB18421	36280	glyceraldehyde 3-phosphate dehydrogenase	3	270
XP_001707988	118760	Glycerol-3-phosphate dehydrogenase	20	1351
XP_001705977	104765	Glycogen phosphorylase	40	2940
XP_001706081	41498	Kinase, NEK	2	107
XP_001707118	35453	Malate dehydrogenase	3	267
AAC47396	61459	malic enzyme	32	2149
XP_001709257	46955	NADH oxidase	15	1057
AAL59603	51439	NADH oxidase	13	781
P28724	49715	RecName: Full=NADP-specific glutamate dehydrogenase	20	1945
XP_001709979	36394	Ornithine carbamoyltransferase	23	1484
CAC33857	21920	ORF-C4	14	885
AAO31976	56389	phosphoacetylglucosamine mutase	4	204
XP_001706090	25742	Protein disulfide isomerase PDI1	7	632
XP_001709256	112759	Protein 21.1	3	201
XP_001709481	87536	Protein 21.1	2	149
XP_001708927	66176	Protein 21.1	9	683
XP_001704344	64147	Protein 21.1	13	1003
XP_001704817	59732	Protein 21.1	4	261
XP_001709808	57078	Protein 21.1	12	961
XP_001708591	88378	Purine nucleoside phosphorylase lateral transfer candidate	6	333
XP_001708948	131700	Pyruvate-flavodoxin oxidoreductase	17	882
AAC47168	97563	pyruvate,phosphate dikinase	11	671
XP_001707507	59907	Pyrophosphate-fructose 6-phosphate 1-phosphotransferase alpha subunit	5	219
XP_001705797	20823	Ribosomal protein L9	2	137
XP_001704829	22839	Ribosomal protein L13a	5	301
XP_001705001	20552	Ribosomal protein L18a	2	143
XP_001707248	30374	Ribosomal protein S4	2	94
XP_001705221	15355	Ribosomal protein S10B	3	214

Accession number	Mass	Description	Peptides	Mascot score
XP_001706152	15698	Ribosomal protein S14	2	183
XP_001704088	17443	Ribosomal protein S18	4	177
XP_001704614	77497	Transketolase	11	681
XP_001706983	76693	VSP with INR	8	399
XP_001706046	20820	Wos2 protein	4	193
XP_001708895	24005	Hypothetical protein GL50803_135885	8	445
XP_001710099	109857	Hypothetical protein GL50803_14584	2	115
XP_001707432	213593	Hypothetical protein GL50803_9183	70	5227
XP_001709157	21146	Hypothetical protein GL50803_7244	2	75
