# Supplementary material for: Ubiquitination dynamics in the early-branching eukaryote Giardia intestinalis
Source: Microbiologyopen. 2013 Apr 23;2(3):525–39. doi: 10.1002/mbo3.88 (PMC3684764; doi:10.1002/mbo3.88)
Supplement: Supplementary file 6 [file mbo30002-0525-SD6.pdf]

Supplementary Table 3

Sequences analysis of protein kinases identified in the *in vitro* ubiquitination reaction			
**Accesion number**	**Descripción**	**Results of BLAST, Pfam and SMART analysis**	**Encystation time**
XP_001704895	Kinase	**Pfam:** Kinase domain (1.9e-38) and 8 ankyrin motifs<br>**BAST:** Homology with protein kinases of CAMK family (*Trichomonas vaginalis* 1e-27)	6, 12 y 24 hours
XP_001709026	Kinase, NEK	**Pfam:** Kinase domain (6.5e-26) and 14 ankyrin motifs	0 hours
XP_001707221	Kinase, NEK	**Pfam:** Kinase domain (2.1e-20) and 9 ankyrin motifs	48 hours
XP_001707357	Kinase, NEK	**Pfam:** Kinase domain (1.4e-39) and 10 ankyrin motifs	0, 6, 12 y 24 hours
XP_001704268	Kinase, NEK	**Pfam:** Kinase domain (2e-57) and 4 ankyrin motifs<br>**Pfam:** CtIP_N domain (2.5e-02), N-terminal domain of CtIP protein. CtIP is a predominantly nuclear protein that interact with BRAC1 E3-ligase and BRCA1-associated RING domain protein (BARD1). CtIP particip in transcriptional regulation, DNA repair, and control of cellular cycle check points.	6 hours
XP_001708517	Kinase, NEK	**Pfam:** Kinase domain (1.7e-36)	6 hours
XP_001706081	Kinase, NEK	**Pfam:** Kinase domain (1.1e-68)	0 hours
XP_001709229	Kinase, NEK	**Pfam:** Kinase domain (1.4e-39)	24 hours
XP_001708873	Kinase, NEK	**Pfam:** Kinase domain (5.2e-45)	0 hours
XP_001710036	Kinase, NEK	**Pfam:** Kianse domain (2.8e-36)	0, 6, 12 y 48 hours
XP_001709632	Kinase, NEK-frag	**Pfam:** Kinase domain (1.8e-20) and 9 ankyrin motifs	0 hours
XP_001705714	Kinase, CDC7	**Pfam:** Kinase domain (2.3e-22)	0, 12 y 24 hours
XP_001705269	Kinase, CAMK CAMKL	**Pfam:** Kianse domain (2.6e-63)<br>**BLAST:** Homology with CBL-interacting protein kinase 3 proteins (CIPK3) (*Arabidopsis lyrata* 2e-53). CIPK3 interact with Calcineurina B like (CBL) and have function in calcium signalling in Arabidopsis.	0 hours
XP_001704849	Kinase, CMGC DYRK	**Pfam:** Kinase domain (3.1e-39)	6 hours
XP_001706920	Kinase, CMGC CK2	**Pfam:** Kinase domain (4.7e-47)<br>**BLAST:** Homology with Casein kinase II proteins (*Candida tropicalis* 1e-76)	0 y 48 hours
XP_001709931	Kinase, CMGC CDK	**Pfam:** Dominio Kinasa (6e-74)<br>**BLAST:** Homology with Cdc2 cyclin-dependent kinase (Cdk1) proteins (*Xenopus laevis* 2e-94)	0 hours
XP_001704058	Kinase, CMGC CDK	**Pfam:** Kinase domain (1.5e-71)<br>**BLAST:** Homology with Cdc2 cyclin-dependent kinase (Cdk1) proteins (*Nicotiana tabacum* 1e-106)	0 hours
XP_001705776	Kinase, PLK	**Pfam:** Kinase domain (5.8e-64) **Pfam:** Two POLO Box domains (1.6e-09 y 7.3e-07) Polo o Polo-like kinasas tienen múltiples papeles durante el ciclo celular.<br>**BLAST:** Homology with Polo-like kinase proteins (*Rattus norvegicus* 2e-70)	0 hours
XP_001707209	Kinase, SCY1	**BLAST:** Homology with SCY1-like 2 CVAK104 proteins (*Acyrthosiphon pisum* 8e-12): CVAK104 in involved in intracellular transport with SNAREs complex	0 hours

Sequences analysis of protein 21.1 identified with the *in-vitro* ubiquitination reaction			
Accesion number	Descripción	Results of BLAST, Pfam and SMART analysis	Encystation time
XP_001710268	Proteína 21.1	**Pfam:** Six ankyrin motifs.	0, 6, 12 y 48 hours
XP_001710302	Proteína 21.1	**Pfam:** Six ankyrin motifs.	0, 12 y 48 hours
XP_001705792	Proteína 21.1	**Pfam:** Five ankyrin motifs.<br>**BLAST:** Homology with Cortactin-binding protein-2 (*H.sapiens* 2e-11). The binding cortactin proteins complex and cortactin regulate the cytoeskeleton actin polymerization.	0 hours
XP_001709256	Proteína 21.1	**Pfam:** Five ankyrin motifs.<br>**BLAST:** Homology with the spindle pole body component (Saccharomyces cerevisiae 7.8e-02).	6 hours
XP_001705064	Proteína 21.1	**Pfam:** Eleven ankyrin mitifs.	0, 6, 12 y 24 hours
XP_001705461	Proteína 21.1	**Pfam:** Six ankyrin motifs.<br>**Pfam:** BLOC1_2 (3.4e-03) - Biogenesis of lysosome-related organelles complex-1 subunit 2 .	0 hours
XP_001709481	Proteína 21.1	**Pfam:** Three ankyrin motifs.<br>**Pfam:** BRE1 (2.1e-04). BRE1 E3-Ubiquitin ligase - Histone H2B mono-ubiquitination.	48 hours
XP_001708876	Proteína 21.1	**Pfam:** Three ankyrin motifs.<br>**BLAST:** COG5243-HRD1. HRD1 protein is a E3-ligase HRD component. HRD is involved in ER-associated degradation system (ERAD).	48 hours
XP_001704344	Proteína 21.1	**Pfam:** Seven ankyrin motifs.	0 y 12 hours
XP_001704817	Proteína 21.1	**Pfam:** Eight ankyrin motifs.	48 hours
XP_001708396	Proteína 21.1	**Pfam:** Seven ankyrin motifs.	6 y 48 hours
XP_001704622	Proteína 21.1	**Pfam:** Four ankyrin motifs.	48 hours

Sequence analysis of Hypothetical proteins identified with the *in-vitro* ubiquitination reaction			
Accession number	Description	Results of BLAST, Pfam and SMART analysis	Encystation time
XP_001705438	Hypothetical protein GL50803_115478	**Pfam/SMART:** Nine Ankyrin repeats.<br>**Pfam:** Myosin tail1 (7.7e-04)<br>**NCBI Anotation:** Ankyrin and SMC_prok_A - chromosome segregation protein SMC.	0 and 48 hours
XP_001707402	Hypothetical protein GL50803_101278	Domains not detected	0, 12 and 24 hours
XP_001708344	Hypothetical protein GL50803_94463	Domains not detected	6 and 48 hours
XP_001706533	Hypothetical protein GL50803_94117	**Pfam:** zf-DBF (1.7e-03) Zinc finger domain	0, 6, 12 and 24 hours
XP_001706938	Hypothetical protein GL50803_28699	Domains not detected	0 hours
XP_001707013	Hypothetical protein GL50803_24451	**Pfam:** FGF Domain (8.2e-06). Fibrobast growt factor family, proteins involved in growth and differentiation in a wide range of contexts.<br>**NCBI Anotation:** FGF- Acidic and basic fibroblast growth factor family.	48 hours
XP_001707206	Hypothetical protein GL50803_23017	**SMART/Pfam:** FAD Domain 1.96e-08 (45 to 95 aa) The forkhead-associated (FHA) domain is a phosphopeptide recognition domain found in many regulatory proteins.	0 and 48 hours
XP_001707920	Hypothetical protein GL50803_22543	Domains not detected	0 hours
XP_001706898	Hypothetical protein GL50803_21628	**Pfam:** UPF0227 (8.9e-02). Despite being classed as uncharacterised proteins, the members of this family are almost certainly enzymes that are distantly related to the alpha/beta hydrolase fold.	0 hours
XP_001708629	Hypothetical protein GL50803_17400	**Pfam:** Cyclin (3.4e-17)	0 hours
XP_001706499	Hypothetical protein GL50803_17332	**Pfam:** DUF2890 (0.41). Domain of unknown finction. This family is conserved in dsDNA adenoviruses of vertebrates.	0 hours

Accession number	Description	Results of BLAST, Pfam and SMART analysis	Encystation time
XP_001704754	Hypothetical protein GL50803_16794	**Pfam:** DUF814 (2.4e-15). Domain of unknown function. This domain occurs in proteins that have been annotated as Fibronectin/fibrinogen binding protein by similarity.	0 hours
XP_001707556	Hypothetical protein GL50803_16653	**Pfam:** FYVE zinger domain (2.7e-16). FYVE-type domains are divided into two known classes: FYVE domains that specifically bind to phosphatidylinositol 3-phosphate in lipid bilayers and FYVE-related domains of undetermined function. Most FYVE domains target proteins to endosomes by binding specifically to phosphatidylinositol-3-phosphate at the membrane surface.	24 hours
XP_001707576	Hypothetical protein GL50803_16648	**NCBI Anotation:** Ankyrin and SMC_prok_A - chromosome segregation protein SMC	48 hours
XP_001707037	Hypothetical protein GL50803_16424	**Pfam:** Mlf1IP (3.1e-04) Is the conserved central region of a group of proteins that are putative transcriptional repressors. The structure contains a putative 14-3-3 binding motif involved in the subcellular localisation of various regulatory molecules, and it may be that interaction with the transcription factor DREF could be regulated through this motif. DREF regulates proliferation-related genes in Drosophila.	48 hours
XP_001709409	Hypothetical protein GL50803_16353	**SMART:** Transmembrane segment (C-terminal)	0, 6, 12 and 24 hours
XP_001704054	Hypothetical protein GL50803_16312	**Pfam:** A2_M-N2 (4.4e-02). Alpha-2-macroglobulin family N-terminal region. The alpha-macroglobulin (aM) family of proteins includes protease inhibitors. **NCBI Anotation:** putative PEP-CTERM system TPR-repeat lipoprotein; TIGR02917	0 hours
XP_001707019	Hypothetical protein GL50803_11380	Domains not detected	0 and 12 hours
XP_001706436	Hypothetical protein GL50803_9861	Domains not detected	0, 6, 12 and 24 hours
XP_001707432	Hypothetical protein GL50803_9183	**Pfam:** MtfA (9.4e-02). MtfA (earlier known as YeeI) is a transcription factor A that binds Mlc (make large colonies), itself a repressor of glucose and hence a protein important in regulation of the phosphoenolpyruvate:glucose-phosphotransferase (ptsG) system, the major glucose transporter in E.coli.	24 hours
XP_001708788	Hypothetical protein GL50803_9098	**Pfam:** MIP-T3 (1.8e-08) Microtubule-binding protein. This protein, which interacts with both microtubules and TRAF3 (tumour necrosis factor receptor-associated factor 3), is conserved from worms to humans.	0 hours
XP_001709369	Hypothetical protein GL50803_8770	**Pfam/NCBI Anotation:** UDG (6.3e-05) Uracil DNA glycosylase superfamily. Uracil-DNA glycosylases are DNA repair enzymes that excise uracil residues from DNA by cleaving the N-glycosylic bond, initiating the base excision repair pathway.	0 and 12 hours
XP_001704451	Hypothetical protein GL50803_8692	**Pfam:** CBS domain (4.2e-04 and 3.1e-07) CBS domains are small intracellular modules that pair together to form a stable globular domain. Pairs of these domains have been termed a Bateman domain. CBS domains have been shown to bind ligands with an adenosyl group such as AMP, ATP and S-AdoMet. CBS domains are found attached to a wide range of other protein domains suggesting that CBS domains may play a regulatory role making proteins sensitive to adenosyl carrying ligands. **NCBI Anotation:** "TIM_phosphate_binding" TIM barrel proteins share a structurally conserved phosphate binding motif and in general share an eight beta/alpha closed barrel structure.	0 and 6 hours
XP_001706561	Hypothetical protein GL50803_8528	Domains not detected	0, 12 and 24 hours
XP_001709157	Hypothetical protein GL50803_7244	**SMART:** Transmembrane segments (3)	6 hours
XP_001704720	Hypothetical protein GL50803_6617	**SMART:** Transmembrane segments (C-terminal) **Pfam:** DDOST_48KDa (1). Members of this family are involved in asparagine-linked protein glycosylation. In particular, dolichyl-diphosphooligosaccharide-protein glycosyltransferase (DDOST), also known as oligosaccharyltransferase EC:2.4.1.119, transfers the high-mannose sugar GlcNAc(2)-Man(9)-Glc(3) from a dolichol-linked donor to an asparagine acceptor in a consensus Asn-X-Ser/Thr motif.	0 hours
XP_001705314	Hypothetical protein GL50803_6171	Domains not detected	0, 6 and 12 hours
XP_001707179	Hypothetical protein GL50803_5800	**Pfam/NCBI Anotation:** START Domain (2.2e-10). START (STeroidogenic Acute Regulatory (STAR) related lipid Transfer) Domain. These domains are 200-210 amino acid in length and occur in proteins involved in lipid transport (phosphatidylcholine) and metabolism, signal transduction, and transcriptional regulation.	0 hours
XP_001704815	Hypothetical protein GL50803_4595	Domains not detected	0 and 24 hours
