# Supplementary material for: Ubiquitination dynamics in the early-branching eukaryote Giardia intestinalis
Source: Microbiologyopen. 2013 Apr 23;2(3):525–39. doi: 10.1002/mbo3.88 (PMC3684764; doi:10.1002/mbo3.88)
Supplement: Supplementary file 7 [file mbo30002-0525-SD7.pdf]

Supplementary Table 4
Ubiquitinations sites identified in the in vitro/in vivo analysis

Accession Number	Protein	Sequence	Modifications	Δ Score	Charge	m/z [Da]	MH+ [Da]	ΔM [ppm]	RT [min]	Ions Matched
XP_001706718.1	ubiiquitin	LIFSGkQLEDNR	K6(GlyGly)	0,60	2	767,40563	1533,80398	1,26	27,55	15/22
XP_001704310.1	Alpha-1 giardin	LAAYLINCACNDk	K13(GlyGly)	0,37	2	763,36168	1525,71608	1,48	28,69	17/34
XP_001704713.1	Dynein heavy chain	EFELkK	K5(GlyGly)	0,16	2	454,24865	907,49003	1,80	39,42	7/10
XP_001704529.1	Elongation factor 1-alpha	MVPQkPLCCETFNDYAPLGR	K5(GlyGly)	0,63	2	1198,56735	2396,12742	3,23	30,58	15/38
XP_001708346.1	Coiled-coil protein	KkTEDFPKELAR	K2(GlyGly)	0,07	2	788,42108	1575,83488	-8,96	29,49	15/22
XP_001708480.1	Kinase, NEK	EGGmkDK	M4(Oxidation) K5(LeuArgGlyGly)	0,21	2	582,29146	1163,57564	-7,00	22,79	7/12
XP_001708446.1	Alpha-SNAP	QAEVkLK	K5(GlyGly)	0,05	2	465,27505	929,54283	1,43	21,52	8/12
XP_001708162.1	Hypothetical protein	RMAkSLITPR	K4(GlyGly)	0,38	2	643,87120	1286,73513	-0,85	29,73	11/18
XP_001705756.1	Hypothetical protein	AKAQkAAK	K5(GlyGly)	0,12	2	465,27564	929,54400	-9,40	21,67	11/14
