# Supplementary material for: Ubiquitination dynamics in the early-branching eukaryote Giardia intestinalis
Source: Microbiologyopen. 2013 Apr 23;2(3):525–39. doi: 10.1002/mbo3.88 (PMC3684764; doi:10.1002/mbo3.88)
Supplement: Supplementary file 8 [file mbo30002-0525-SD8.pdf]

Supplementary Table 5
Edogenous proteins from trophozoites purified by immunoprecipitation

Immunoprecipitation antibody FK2					
**Acssesion Number**	**Mass**	**Peptides**	**Mascot score**	**Description**	**Identified in in-vitro reaction**
XP_001705755.1	64072	2	105	Arginine deiminase	YES
XP_001709475.1	74296	9	598	Bip	
gi|159116020	33839	2	164	Carbamate kinase	
AAA19122.1	71288	7	476	Cytoplasmic 70 kDa heat shock protein	YES
XP_001704962	79444	11	646	Dynamin	YES
AAB18421	36280	3	225	Glyceraldehyde 3-phosphate dehydrogenase	YES
BAJ33526	80770	12	660	Heat shock protein 90	
gi|159113998	36450	2	165	Hypothetical protein GL50803_115337	
XP_001706898.1	42334	3	245	Hypothetical protein GL50803_21628	YES
XP_001706436.1	42667	6	319	Hypothetical protein GL50803_9861	YES
XP_001707782.1	50357	5	385	Protein disulfide isomerase PDI2	YES
XP_001707224.1	26688	2	78	Ribosomal protein S2	YES
XP_001709079.1	43227	6	414	Translation initiation factor eIF-4A, putative	
XP_001707536	52357	6	363	Transglutaminase/protease, putative	
XP_001707422.1	75433	2	310	VSP	
CAA49657.1	8597	6	402	Ubiquitin	YES
gi|159113055	28540	2	133	14-3-3 protein	YES
**Immunoprecipitation antibody anti-Lys48**					
**Acssesion Number**	**Mass**	**Peptides**	**Mascot score**	**Description**	**Identified in in-vitro reaction**
XP_001710238.1	97053	2	87	Alcohol dehydrogenase	YES
XP_001705755.1	64072	8	461	Arginine deiminase	YES
AAN78305.1	48858	2	216	Alpha-tubulin	YES
XP_001709357.1	236173	13	986	Axoneme-associated protein GASP-180	
XP_001709475.1	74296	12	882	Bip	
AAA19122.1	71288	7	528	Cytoplasmic 70 kDa heat shock protein	YES
XP_001705159.1	25386	2	139	GTP-binding nuclear protein RAN/TC4	
XP_001704962	79444	18	1109	Dynamin	YES
BAA06215.1	90464	2	63	Elongation factor 2	YES
XP_001709336.1	48171	3	210	Enolase	
XP_001708232.1	33839	7	430	Carbamate kinase	
AAB18421.1	36280	6	360	Glyceraldehyde 3-phosphate dehydrogenase	YES
BAJ33526.1	80770	26	1666	Heat shock protein 90	
XP_001705344.1	72703	21	1457	High cysteine membrane protein Group 5	
EFO65550.1	12530	2	149	Hypothetical protein GLP15_1799	YES
XP_001705592.1	14472	2	148	Hypothetical protein GL50803_5810	
XP_001705607.1	17265	2	90	Hypothetical protein GL50803_16404	
XP_001706898.1	42334	2	93	Hypothetical protein GL50803_21628	YES
XP_001706436.1	42667	2	134	Hypothetical protein GL50803_9861	YES
XP_001709409	83582	2	57	Hypothetical protein GL50803_16353	YES
XP_001707556	103032	3	133	Hypothetical protein GL50803_16653	YES
XP_001708448.1	127578	7	530	Importin beta-3 subunit	
XP_001707838.1	18014	2	135	Peptidyl-prolyl cis-trans isomerase B precursor	
XP_001707782.1	50357	7	616	Protein disulfide isomerase PDI2	YES
XP_001706397.1	76941	2	140	Protein 21.1	YES
BAB58887	20718	2	168	Rab-like protein A	
XP_001707035	24410	2	69	RER1-like protein-retention of ER proteins	
XP_001705797.1	20823	2	104	Ribosomal protein L9	YES
XP_001705020.1	21720	3	152	Ribosomal protein S7	
XP_001705652.1	14721	3	121	Ribosomal protein S15A	
XP_001707031.1	31659	2	175	Ser/Thr protein kinase	
XP_001704098	71705	3	195	Ser/Thr phosphatase 2A, 65kDa reg sub A	
AAD51093.1	22464	6	319	Thioredoxin peroxidase homolog	YES
XP_001707536.1	52357	2	65	Transglutaminase/protease, putative	
XP_001709079.1	43227	4	259	Translation initiation factor eIF-4A, putative	
XP_001709528.1	88582	4	903	VSP	
XP_001708498.1	63009	2	144	VSP AS8	
CAA49657.1	8597	6	442	ubiquitin	YES
XP_001706046.1	20820	3	182	Wos2 protein	
XP_001706755.1	28540	2	113	14-3-3 protein	YES
